# An updated and comprehensive review on the ethnomedicinal uses, phytochemistry, pharmacological activity and toxicological profile of *Tinospora crispa* (L.) Hook. f. & Thomson

**DOI:** 10.1007/s11101-022-09843-y

**Published:** 2022-11-03

**Authors:** Ehfazul Haque, Md. Sazzadul Bari, Labony Khandokar, Juhaer Anjum, Ibrahim Jantan, Veronique Seidel, Md. Areeful Haque

**Affiliations:** 1 Department of Medicinal Chemistry, College of Pharmacy, University of Minnesota, Minneapolis, MN 55455 USA; 2 Department of Chemistry, Purdue University, West Lafayette, IN 47907 USA; 3 Department of Pharmacy, East West University, Dhaka, 1212 Bangladesh; 4 Department of Pharmacy, Faculty of Pharmacy, University of Dhaka, Dhaka, 1000 Bangladesh; 5 Institute of Systems Biology (INBIOSIS), Universiti Kebangsaan Malaysia, UKM, Bangi, Selangor Malaysia; 6 Natural Products Research Laboratory, Strathclyde Institute of Pharmacy and Biomedical Sciences, University of Strathclyde, Glasgow, UK; 7 Department of Symptom Research, The University of Texas MD Anderson Cancer Center, Houston, TX 77030 USA; 8 Department of Pharmacy, International Islamic University Chittagong, Chittagong, 4318 Bangladesh

**Keywords:** *Tinospora crispa*, Ethnomedicinal uses, Phytoconstituents, Pharmacological activity, Toxicological profile

## Abstract

*Tinospora crispa* (L.) Hook. f. & Thomson (Menispermaceae) is a plant indigenous to Africa and South-East Asia. It is widely used in ethnomedicine to alleviate various diseases including hypertension, diabetes, rheumatism, jaundice, inflammation, fever, fractures, scabies, and urinary disorders. A total of 167 phytoconstituents, belonging to 12 different chemical categories, including alkaloids, flavonoids, terpenoids, and phenolic compounds have thus far been isolated from various parts of *T. crispa.* Numerous in vitro and in vivo investigations have already established the antidiabetic, anticancer, antiparasitic, antimicrobial, immunomodulatory, hepatoprotective, analgesic, antipyretic, antihyperuricemic, and pesticidal activity of this plant, as well as its effects on the cardiac and the central nervous system. Most pharmacological investigations to date have been carried out on plant extracts and fractions. The exact identity of the phytoconstituents responsible for the observed biological effects and their mode of action at the molecular level are yet to be ascertained. Toxicological studies have demonstrated that *T. crispa* is relatively safe, although dose-dependent hepatotoxicity is a concern at high doses. This review presents a comprehensive update and analysis on studies related to the ethnomedicinal uses, phytochemistry, pharmacological activity and toxicological profile of *T. crispa*. It provides some critical insights into the current scientific knowledge on this plant and its future potential in pharmaceutical research.

## Introduction

*Tinospora crispa* (L.) Hook. f. & Thomson is a deciduous climbing plant belonging to the Menispermaceae family. It is native to the tropical rainforests and mixed deciduous forests of Africa and South-East Asia (Pathak et al. 1995). The plant is used ethnomedicinally in several countries, including Bangladesh, Malaysia, China, Philippines, Brunei, Vietnam, Laos, Thailand, Cambodia, Indonesia, Martinique, and Nepal (Quisumbing 1951; Forman 1981; Noor et al. 1989; Longuefosse and Nossin 1996; Ahmad and Ismail 2003; Grenand et al. 2004; Dweck and Cavin 2006; Hout et al. 2006; Li et al. 2006; Roosita et al. 2008; Islam et al. 2011; Rahmatullah et al. 2011; Koay and Koay 2013; Haque et al. 2017; Dapar 2020; Dapar et al. 2020; Paudel et al. 2020). Its leaves, stems, seeds, rhizomes and roots are used in the formulation of various preparations that are employed to treat a range of conditions such as hypertension, diabetes, rheumatism, jaundice, inflammation, fever, malaria, loss of appetite, fractures, scabies, and urinary disorders (Gimlette and Burkill 1930; Quisumbing 1951; Kongsaktrakoon et al. 1984; Noor et al. 1989; Longuefosse and Nossin 1996; Ahmad and Ismail 2003; Hout et al. 2006; Li et al. 2006; Roosita et al. 2008; Rahmatullah et al. 2009; Srithi et al. 2009; Haque et al. 2011; Islam et al. 2011; Koay and Koay 2013; Kadir et al. 2014). The use of *T. crispa* in several of these conditions has already been validated scientifically in in vitro and in vivo studies which have demonstrated the biological (e.g. cardiovascular, hypoglycemic, cytotoxic, immunomodulatory, anti-inflammatory, antimalarial) activity of extracts, fractions, and some phytoconstituents (Noor et al. 1989; Anulukanapakorn et al. 1999; Amom et al. 2011; Ibahim et al. 2011; Praman et al. 2011, 2013; Hipol et al. 2012; Kamarazaman et al. 2012; Lam et al. 2012; Lokman et al. 2013; Abood et al. 2014). The phytoconstituents in *T. crispa* are diverse and Clerodane-type furanoditerpenoids are characteristic for the species (Bisset and Nwaiwu 1983; Pachaly et al. 1992; Umi Kalsom and Noor 1995; Cavin et al. 1998; Kongkathip et al. 2002; Choudhary et al. 2010a, b; Chung 2011; Lam et al. 2012; Praman et al. 2012; Yusoff et al. 2014; Ahmad et al. 2016b). Many studies have focused on the bioactivity of *T. crispa* extracts. Relatively few studies have been carried out on *T. crispa* phytoconstituents. Toxicity and biosafety studies on *T. crispa* phytoconstituents are also scarce. Given the potential of *T. crispa* as a possible source of new drug leads for various pathological conditions, further pharmacodynamic and pharmacokinetic investigations of its phytoconstituents are warranted.

This study aims to provide a detailed account of the taxonomy, phytochemistry, pharmacology, and toxicology relevant to *T. crispa,* so that it may serve as a valuable resource providing future direction for researchers. Electronic versions of tertiary literature sources (e.g. Google Scholar, PubMed, ScienceDirect, Scopus, Wiley Online Library, SpringerLink, Semantic Scholar, Web of Science and MEDLINE) were used to retrieve data on the ethnopharmacology, phytochemistry, pharmacology, and toxicology of *T. crispa* published within 1930–2021.

## Vernacular names

The following vernacular names for *T. crispa* have been reported (Quisumbing 1951; Forman 1981; Noor et al. 1989; Longuefosse and Nossin 1996; Ahmad and Ismail 2003; Grenand et al. 2004; Dweck and Cavin 2006; Hout et al. 2006; Li et al. 2006; Roosita et al. 2008; Islam et al. 2011; Rahmatullah et al. 2011; Koay and Koay 2013; Haque et al. 2017; Dapar 2020; Dapar et al. 2020):

- Bangladesh: Guloncho-ban, Golonchi, Khorosh, Guntai
- India: Dier, Faridbuti, Dagadi, Chipuru-tige, Kattle-ti, Giloya
- Malaysia: Brotowali, Akar Patawali, Patawali, Akar Seruntum, Seruntum, Sapai, Daun akar walli
- China: Da ye ruan jin teng, Bo ye qing, Niu dan, Ye qing niu dan, Fa leng teng
- Philippines: Makabuhay, Panyawan, Meliburigan, Manunggal
- Thailand: Boraphet, Ho-Boraphet, Khruea khao, Pae jae, Wan kab hoi yai, Chung ching, Kuakhohoo, Ching cha li
- Indonesia: Bratawali, Brotowali, Antawali, Andawali, Putrawali, Daun gade
- Cambodia: Banndol Pech
- Vietnam: Day coc
- Laos: Hmab Iab, Kheuah khao, Ho
- Brunei: Ratnawali, Akar nawali, Nawali
- Republic of Guinea (French Guinea): Liane-quinine
- Guyana: Liane amère
- Martinique Island: Lyann span, Zeb kayenn
- Indochina: Day than thong, Bandaul pich, Day ki nin, Thuoc sot ret
- Java: Brotowali, Andawali, Putrowali, Akar pahat

## Taxonomy

**Fig. 1:**
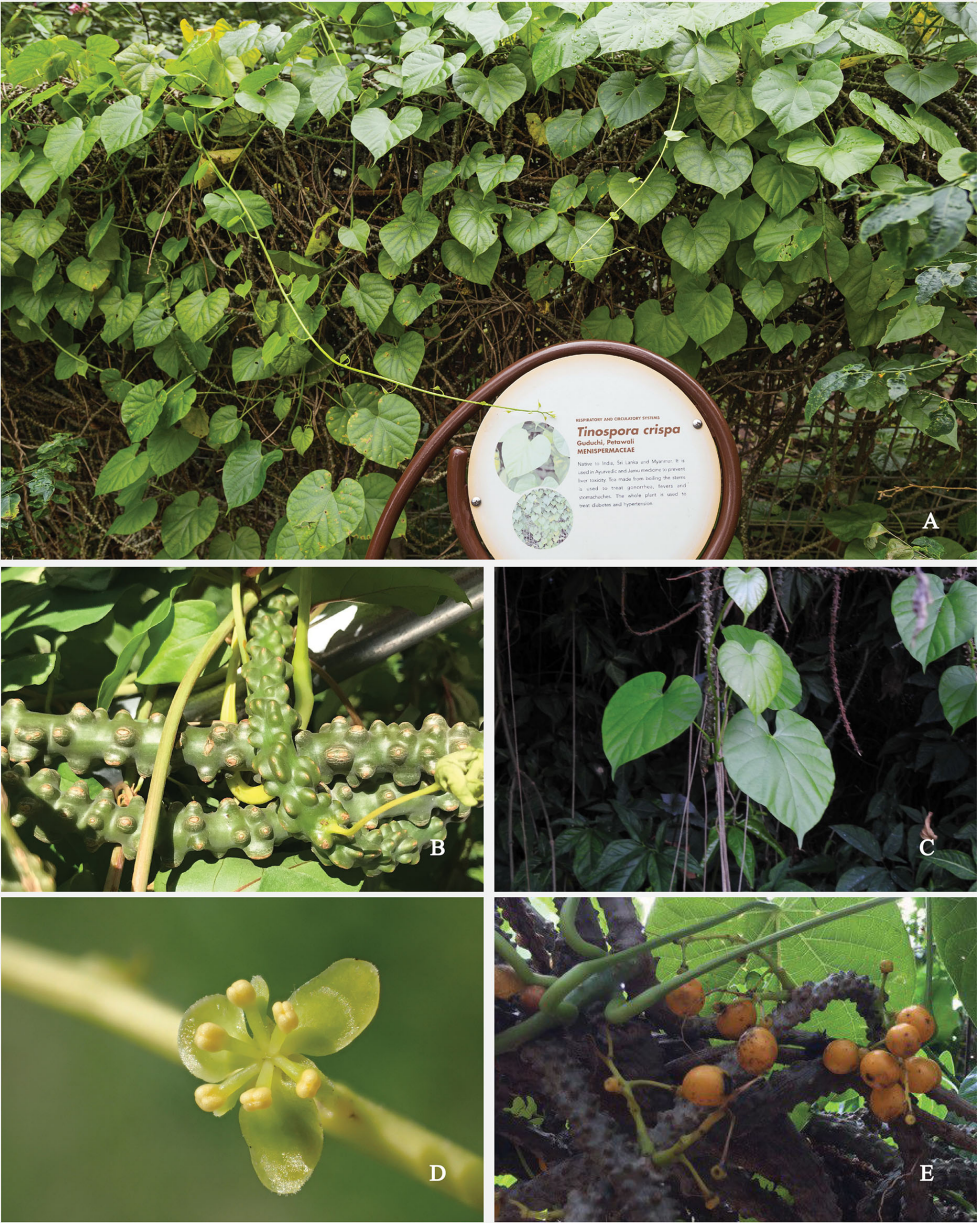
*Tinospora crispa* (L.) Hook. f. & Thomson. **A** Whole plant, **B** Stem, **C** Leaves, **D** Flower, **E** Fruit

*Tinospora crispa* is one of the 34 species that belong to the genus *Tinospora.* All species of this genus are found in tropical and subtropical regions of Asia, Africa and Australia. Most species produce bioactive constituents (especially diterpenoids and alkaloids) and are used widely in ethnomedicine (Chi et al. 2016). *Tinospora crispa* is also known as *Chasmanthera crispa* Baill., *Cocculus crispus* DC., *Cocculus verrucosus* Wall., *Menispermum crispum* L., *Menispermum rimosum* Blanco, *Menispermum tuberculatum* Lam., *Menispermum verrucosum* Roxb., *Menispermum verrucosum* Roxb. ex Fleming, *Tinospora crispa* Diels, *Tinospora gibbericaulis* Hand.-Mazz., *Tinospora mastersii* Diels, *Tinospora rumphii* Boerl., *Tinospora thorelii* Gagnep. and *Tinospora tuberculata* Beumée ex K. Heyne. (The Plant List 2013; Global Biodiversity Information Facility 2021; World Flora Online 2021). This species has a generally fleshy, with older stems being fleshier than younger ones. Younger stems present a thin membranous and glabrous epidermis is characteristic of younger stems, while tubercles are observed on older ones. The stem contains a bitter, milky sap. *Tinospora crispa* has long, filamentous, aerial roots. The leaves are cordiform in shape and are usually 6–12 cm long and 7–12 cm wide. They are marginally fleshy with chartaceous leaf-blades. The dried leaves are quite delicate. Domatia are not usually observed, but a flat pocket appears intermittently in the axis of the basal nerves on the ventral surface. The leaf petioles are 5–15 cm long and glabrous. The flowers are fascicled and greenish-yellow or yellow. The male inflorescences are taller and thinner compared to the female counterparts, 5–10 cm versus 2–6 cm respectively. Both male and female flowers share morphological similarities in terms of sepals and petals with six green sepals in two verticils. The inner three sepals are obovate while the rest are ovate. Both male and female flowers have 3–6 yellow petals. The fruits are vermillion or scarlet, with a pale white endocarp. They are ellipsoidal, 7–8 mm long, and feature a distinctive dorsal ridge with a small ventral aperture and a deeply seed-cavity intrusive condyle. The seeds are curved, bean-shaped, and white. The root is succulent (Forman 1981; Patel et al. 2013; Haque et al. 2017). *Tinospora crispa* and its various parts are illustrated for identification in Fig. 1.

The complete taxonomic classification of *T. crispa* is provided below (Global Biodiversity Information Facility 2021):

Kingdom: Plantae

Division: Magnoliophyta

Class: Magnoliopsida

Order: Ranunculales

Family: Menispermaceae

Genus: Tinospora

Species: Tinospora crispa

## Ethnomedicinal uses

*Tinospora crispa* is used in ethnomedicine predominantly in South-East Asia. Some of its uses are common across multiple ethnicities (e.g. diabetes) while others are reserved to certain regions only. In Bangladesh, various preparations are used for fever, body pain, rheumatism, skin diseases, paralysis, abdominal pain, intestinal disorders and leprosy (Rahmatullah et al. 2009, 2011; Islam et al. 2011; Kadir et al. 2014). In Malaysia, infusions of the stems and of the whole plant are used as a postpartum remedy and to treat type-2 diabetes mellitus, tuberculosis, cholera, malaria, hypertension, lumbago, muscle pain and intestinal parasites (Forman 1981; Noor et al. 1989; Ahmad and Ismail 2003; Mohamad et al. 2011; Dapar 2020). In the Philippines, the stems and leaves are employed for fever, indigestion, flatulence, intestinal disorders, diarrhea, vomiting, ulcer, body ache, rheumatism, toothache, ocular soreness, scabies, lacerations and boils (Quisumbing 1951; Dapar 2020; Dapar et al. 2020). In Thailand, the leaves, stems, roots and seeds are prepared into powders, infusions and decoctions to treat wounds, itches, cholera, diabetes, fever, rheumatism, intestinal parasites, snake-bites, syphilitic sores, sore eyes, and alcohol or drug-induced poisoning (Dweck and Cavin 2006). People in the Yao community in China use hot infusions of the stems as bath water to treat fractures, contusions, furuncles, carbuncles and viper-bites (Li et al. 2006). In China again, the plant is used for fever, septicemia, scabies and ulcers (Koay and Koay 2013). In the South Kerala region of India, locals use the plant as an antidiabetic (Thomas et al. 2016). The use of *T. crispa* as an antimalarial agent is widespread in Malaysia, the Philippines, Indonesia, Vietnam, Southern Laos and the Republic of Guinea (Forman 1981; Ahmad and Ismail 2003; Bertani et al. 2005; Elkington et al. 2014; Ramadani et al. 2018; Dapar 2020; Dapar et al. 2020). Indonesians also employ the plant for hyperglycemia, inflammation, fever and rheumatism. The last two uses are also reported in Cambodia (Hout et al. 2006; Adnan et al. 2016; Ramadani et al. 2018). Apart from the aforementioned uses, *T. crispa* stems are also employed to treat jaundice and fever in Vietnam (Forman 1981). The Kadayan Malay community in the Sengkurong mukim region of Brunei use the stems for hypertension and abdominal ache (Dapar 2020). In Guyana, a bitter beverage produced from *T. crispa* macerated stems, combined with *Quassia amara* bark, is taken for albuminuria and diabetes (Grenand et al. 2004; Thomas et al. 2016). In Martinique, the leaves and stems are used in decoctions and tinctures to treat diabetes (Longuefosse and Nossin 1996). The ethnomedicinal uses of *T. crispa* are listed in Table 1.

**Table 1:** Ethnomedicinal uses of *Tinospora crispa*

Country	Part used	Preparation and method of administration	Ethnomedicinal use (location of use)	References
Bangladesh	Leaves Stem	Juice used topically	Body pain, rheumatism	(Rahmatullah et al. 2009)
		Hot infusion	Skin disease	(Kadir et al. 2014)
	Stem	Pills prepared from pulverized dried stems and honey	Paralysis	(Kadir et al. 2014)
		Juice co-administered with Neem juice (*Azadirachta indica)* and honey	Abdominal pain	
		Juice prepared by maceration	Intestinal disorders (Garo and non-Garo traditional practitioners in Tangail district)	(Rahmatullah et al. 2011)
	Whole Plant	Hot infusion with coconut oil (*Cocos nucifera*)	Leprosy	(Kadir et al. 2014)
	Vines	Aqueous maceration co-administered with sugarcane (*Saccharum officinarum)* molasses	Fever	(Islam et al. 2011)
India	–	–	Diabetes (South Kerala)	(Thomas et al. 2016)
Malaysia	Stem	Hot infusion (administered orally)	Type-II diabetes mellitus	(Noor et al. 1989)
		–	Tuberculosis	(Mohamad et al. 2011)
		Infusion	Vermifuge	(Forman 1981)
	Whole plant	Infusion	Cholera	(Forman 1981)
		Hot infusion (administered orally)	Malaria, hypertension (Kadazan-dusun community)	(Ahmad and Ismail 2003)
	–	–	Hypertension, diabetes, lumbago, muscle pain, postpartum remedy (Murut community in Sabah)	(Dapar 2020)
China	Stem	Hot infusion as medical bath water	Fracture, contusion, furuncle, carbuncle, viper-bites (Yao community)	(Li et al. 2006)
	–	–	Fever, septicemia, tropical ulcer, scabies	(Koay and Koay 2013)
Philippines	Leaves, Stem	Aqueous extract	Indigestion, flatulence, diarrhea, rheumatism	(Quisumbing 1951)
	Stem	Alcohol decoction	Fever, malaria, intestinal disorders, ulcer, diarrhea, vomiting, rheumatism, abortifacient, dysmenorrhea, boils, body ache, toothache	(Dapar 2020; Dapar et al. 2020)
		Sap (topical use)	Ocular Soreness, Scabies, Lacerations	
		Infusion with coconut oil (*Cocos nucifera*) or gasoline	Rheumatism, abdominal problems, flatulence, body ache, abortifacient	
Thailand	Leaves	Crushed powder (topical use)	Wounds	(Dweck and Cavin 2006)
		Poultice	Itch	
	Leaves, Stem, Root	Decoction	Cholera, diabetes, fever, rheumatism, snake-bites	
	Stem	Infusion	Vermifuge	
		Decoction (topical use)	Syphilitic sores, sore eyes	
		Decoction	Hemorrhoid	(Ahmad et al. 2016a)
	Seeds	Cold infusion of powder (administered orally)	Poisoning, drug- or alcohol-induced intoxication	(Srithi et al. 2009)
Indonesia	Stem	Infusion	Hyperglycemia, malaria, rheumatic arthritis, fever, hepatitis	(Ramadani et al. 2018)
	–	–	Malaria, rheumatism, fever, inflammation, diabetes, cholera	(Adnan et al. 2016)
Cambodia	Stem	–	Rheumatism, fever	(Hout et al. 2006)
Vietnam	Stem	Hot infusion (administered orally)	Jaundice, fever	(Forman 1981)
		Dry powder	Malaria	
Laos	Stem, Rhizome	–	Malaria (Southern Laos)	(Elkington et al. 2014)
Brunei	Stem	Ingested orally	Hypertension, diabetes, abdominal ache (Kadayan Malay community in Sengkurong mukim)	(Dapar 2020)
Republic of Guinea (French Guinea)	–	–	Malaria	(Bertani et al. 2005)
Guyana	Stem	Maceration in absinthe, rum or cognac, mixed with the bark of *Quassia amara*	Albuminuria, diabetes	(Grenand et al. 2004; Thomas et al. 2016)
Martinique Island	Leaves	Decoction (administered orally)	Diabetes	(Longuefosse and Nossin 1996)
	Stem	Decoction (administered orally)	Diabetes	
		Tincture (administered orally)	Diabetes	

## Phytoconstituents

**Fig. 2:**
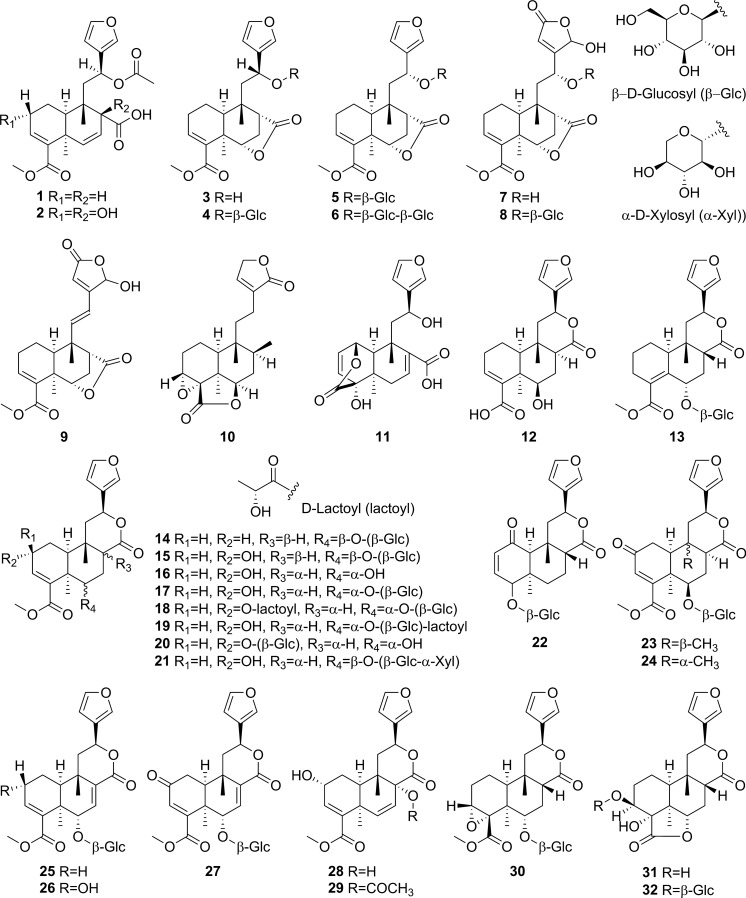

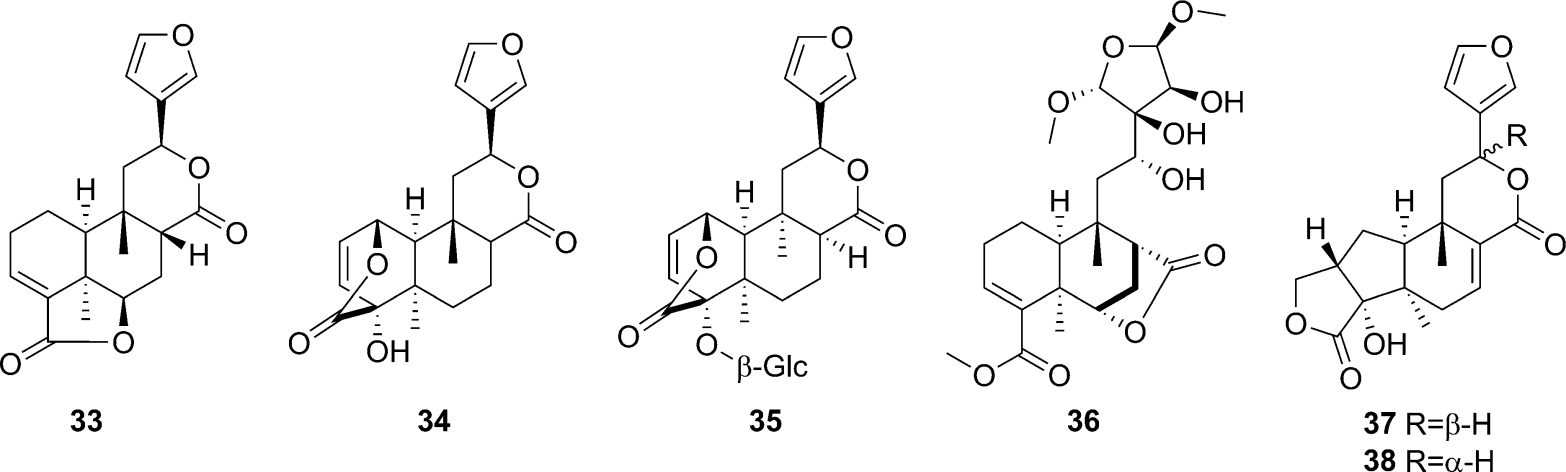
Clerodane-type furanoditerpenoids from *Tinospora crispa*

**Fig. 3:**
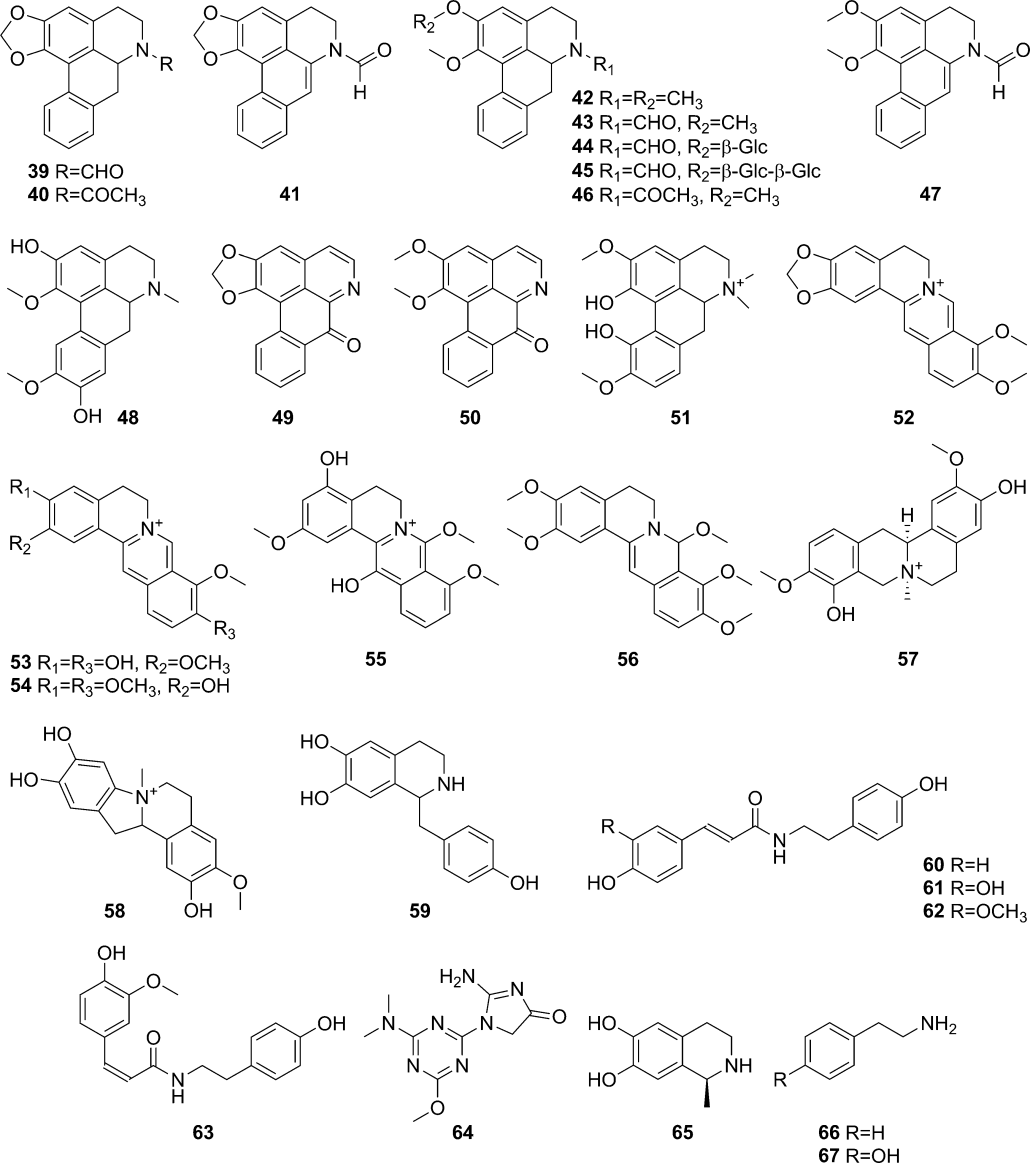
Alkaloids from *Tinospora crispa*

**Fig. 4:**
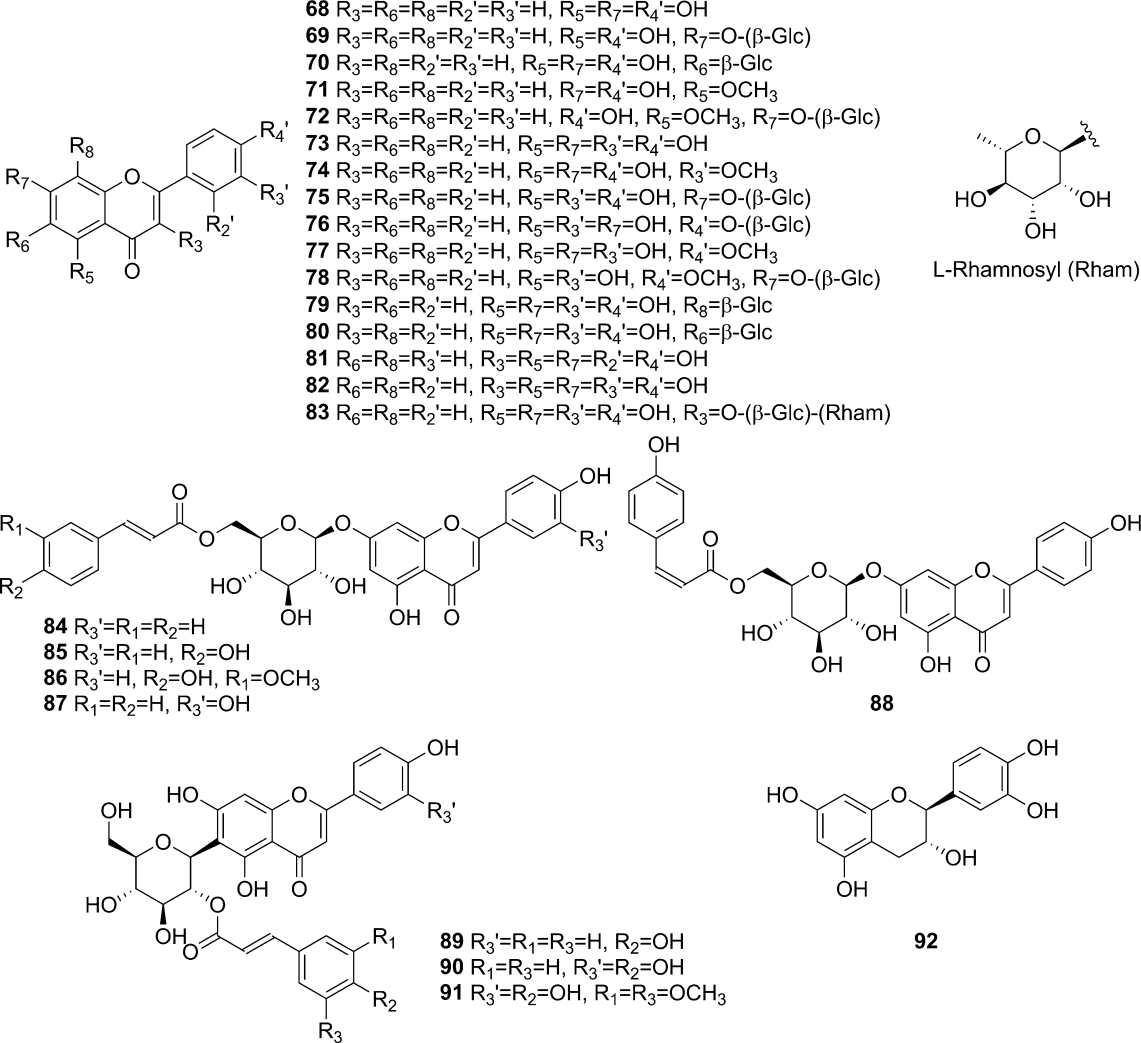
Flavonoids from *Tinospora crispa*

**Fig. 5:**
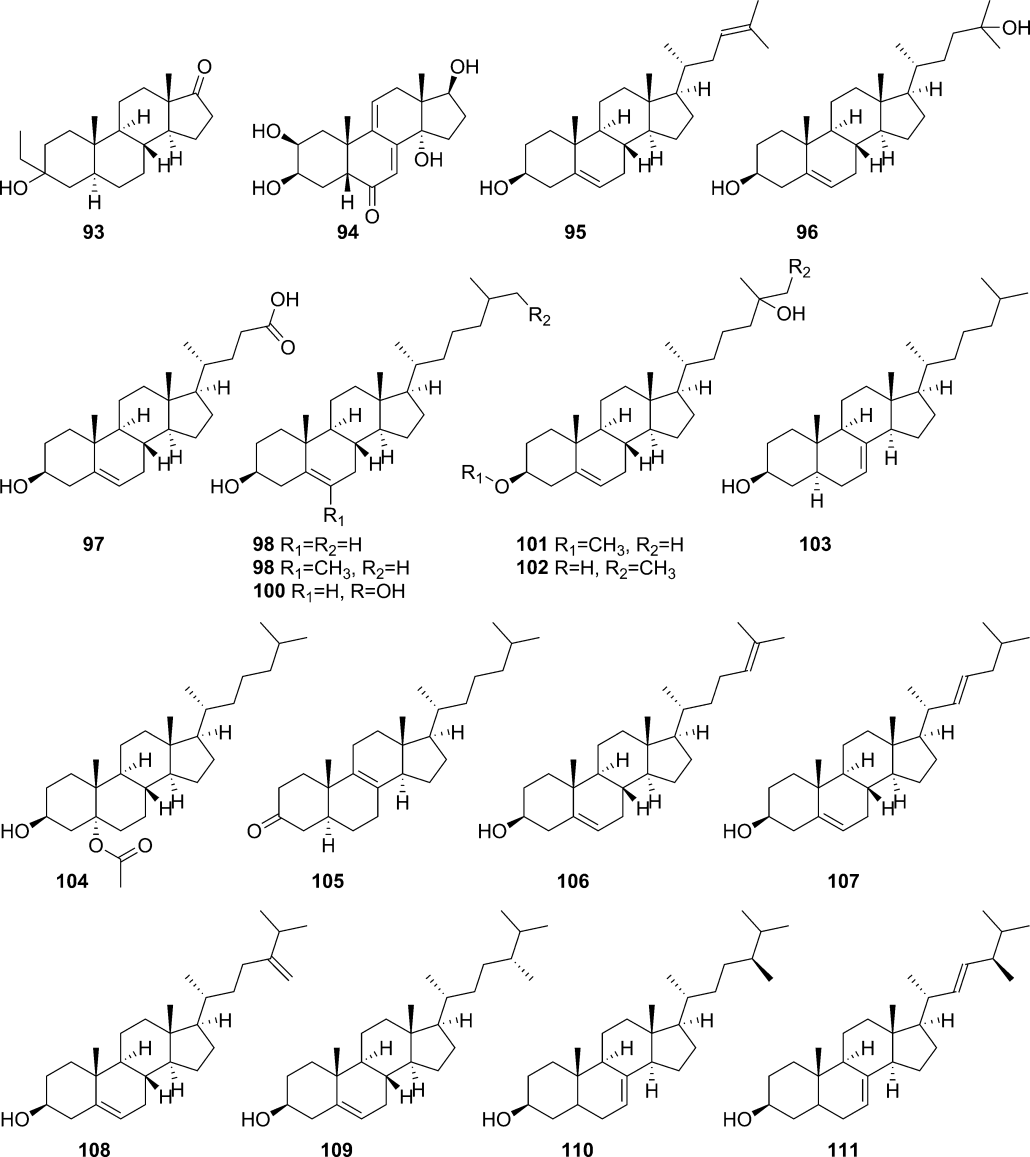

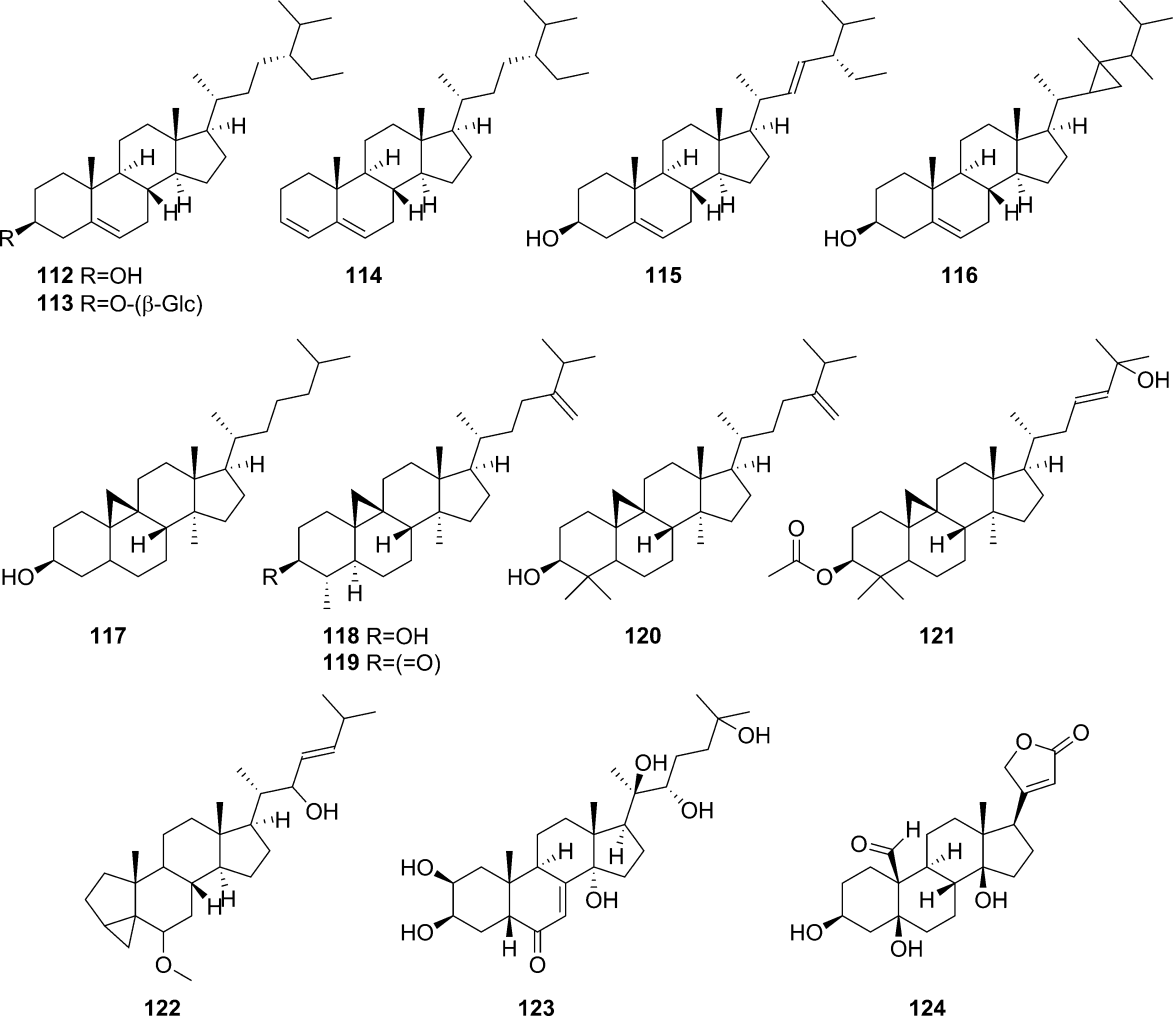
Steroidal compounds from *Tinospora crispa*

**Fig. 6:**
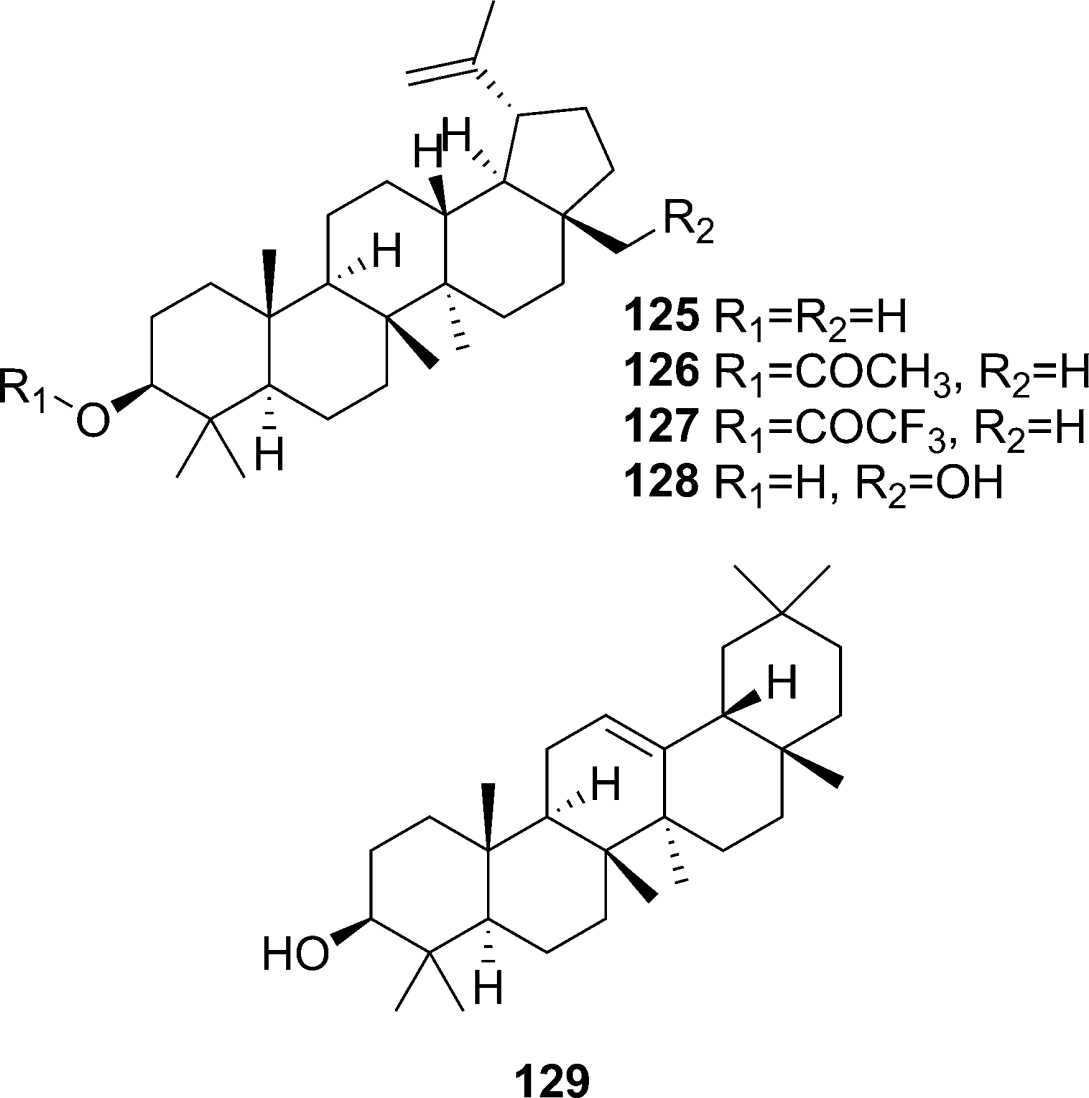
Triterpenes from *Tinospora crispa*

**Fig. 7:**
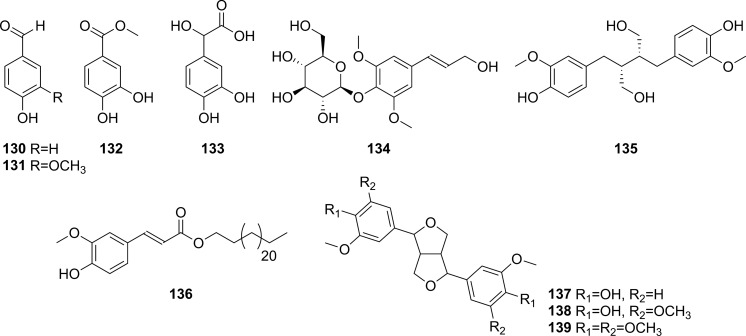
Phenolic compounds from *Tinospora crispa*

**Fig. 8:**
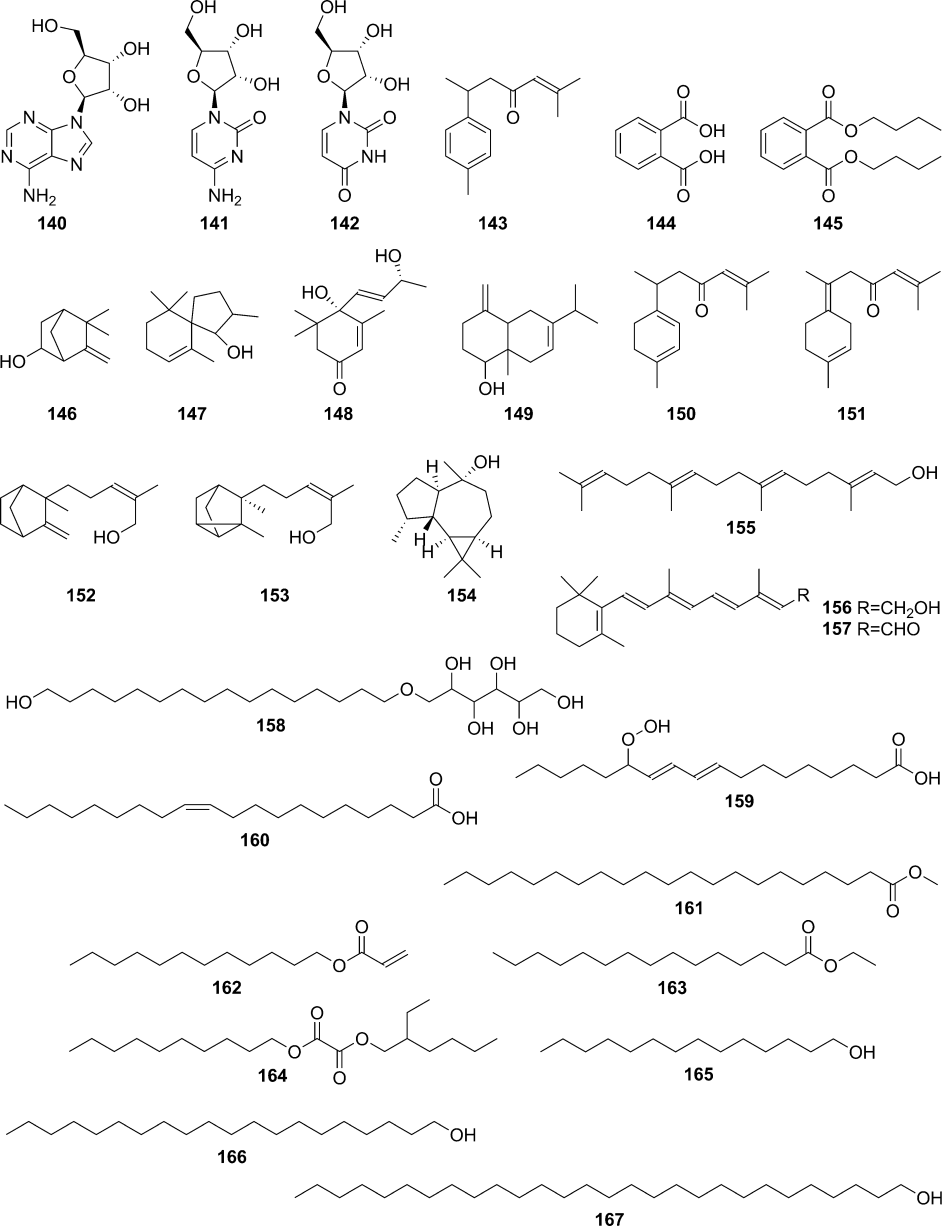
Nucleosides, aromatic, volatile terpenoids and fatty compounds from *Tinospora crispa*

Extensive phytochemical investigations on the aerial parts of *T. crispa,* both as a whole and as individual parts (stems, leaves, and vines), led to the identification of 167 phytoconstituents belonging to diverse chemical classes. Clerodane-type furanoditerpenoids are the most abundant phytoconstituents in this species. A considerable number of alkaloids, flavonoids, and steroidal compounds have also been reported. Other classes of secondary metabolites, present to a lesser extent, include triterpenes, phenolic compounds, nucleosides, aromatic constituents, volatile terpenoids and long-chain fatty acid derivatives. All compounds reported from *T. crispa* to date are listed in Table 2 and their structures are illustrated in Figs. 2, 3, 4, 5, 6, 7 and 8.

**Table 2:** Phytoconstituents and biological activities of *Tinospora crispa*

No	Compounds	Occurrence	Study type	Dose administered	Biological activity	Assays	References
*Clerodane-type furanoditerpenoids*							
1	Crispene C	Stems					(Hossen et al. 2016)
2	Crispene F	Stems			Significant cytotoxicity against STAT3-dependent MDA-MB 231 breast cancer cells	Cell-free fluorescent polarization assay	(Noman et al. 2018)
3	Crispene D	Stems					(Hossen et al. 2016; Noman et al. 2018)
4	Borapetoside E	Stems					(Hossen et al. 2016)
		Stems	In vivo		No inotropic effect on the electrical field-stimulated left atrium	Female Wistar rats	(Praman et al. 2013)
		Stems	In vivo	1–10 mg/kg	No effect on the blood pressure and heart rate in rats	Female Wistar rats	(Praman et al. 2012)
		Vines	In vivo		Improved hyperglycemia, insulin resistance, hepatic steatosis, hyperlipidemia, and increased oxygen consumption. Suppressed the concentration of sterol regulatory element binding proteins (SREBPs) accounting for lipid synthesis in the liver and the adipose tissue	High-fat-diet (HFD)-induced obese mice	(Xu et al. 2017)
		Vines	In vivo	20, 40 and 200 mg/kg	Dose-dependently lowered serum glucose levels	Male ICR and db/db mice	(Gao et al. 2016)
		Vines					(Lam et al. 2012)
5	Rumphioside I	Aerial parts					(Ahmad et al. 2016a)
6	Borapetoside D	Aerial parts		1–100 µM	No visible cytotoxicity	MTT assay on PC-3 (human prostate cancer) and 3T3 (mouse fibroblasts) cells	(Choudhary et al. 2010b)
		Stems	In vivo	1–10 mg/kg	No effect on blood pressure and heart rate	Female Wistar rats	(Praman et al. 2012)
		Vines					(Lam et al. 2012; Gao et al. 2016)
		Stems	In vivo		No inotropic effect on the electrical field-stimulated left atrium	Female Wistar rats	(Praman et al. 2013)
7	Rumphioside F	Vines					(Gao et al. 2016)
8	Tinosporol A	Stems					(Rahman et al. 2020)
		Vines					(Lam et al. 2012; Gao et al. 2016)
9	Tinosporol C	Vines					(Gao et al. 2016)
10	Crispene E	Stems			Significant cytotoxicity against STAT3-dependent MDA-MB 231 breast cancer cells	Cell-free fluorescent polarization assay	(Mantaj et al. 2015)
11	Crispene G	Stems			Significant cytotoxicity against STAT3-dependent MDA-MB 231 breast cancer cells	Cell-free fluorescent polarization assay	(Noman et al. 2018)
12	Crispene B	Stems					(Hossen et al. 2016)
13	Cordioside	Aerial parts					(Ahmed et al. 2006)
		Stems					(Abood et al. 2014)
14	Borapetoside C (Tinocrisposide)	Aerial parts	In vitro	1–100 µM	No visible cytotoxicity	MTT assay on PC-3 (human prostate cancer) and 3T3 (mouse fibroblasts) cells	(Choudhary et al. 2010b)
		Stems					(Langrand et al. 2014; Parveen et al. 2019)
		Stems	In vivo	500 mg/kg	No apparent hepatotoxicity	Male ND-4 mice	(Parveen et al. 2020)
		Stems	In vitro	100, 200, 400, 600, 800, and 1000 µg/mL	Significant anti-inflammatory activity and no hemolytic activity	Human red blood cell (HRBC) membrane stabilization assay	(Adnan et al. 2019)
		Stems	In vitro		No discernible cytotoxicity on H1299 and MCF-7 cells (IC_50_ values of 70.9 µg/mL > 100 µg/mL, respectively)	MTT assay	(Adnan et al. 2016)
		Vines	In vitro		Remarkable inhibition of α-glucosidase and α-amylase (IC_50_ values of 0.527 and 0.775 mg/mL, respectively)	Enzyme inhibitory assay	(Hamid et al. 2015)
		Vines	In vivo	5 mg/kg	Remarkably lowered serum glucose levels and reduced hepatic gluconeogenesis as well as up regulated glucose use	Streptozotocin (STZ)-induced type-1 diabetic mice (T1DM) in ICR mice	(Lam et al. 2012)
		Vines	In vivo	5 mg/kg	Augmented insulin sensitivity and glucose uptake, and decreased plasma glucose levels as well as the development of insulin resistance in normal and type-2 DM (T2DM) mice	Male ICR mice	(Ruan et al. 2012)
15	(2R,5R,6R,8R,9S,10S,12S)-15,16-Epoxy-2-hydroxy-6-*O*-(β-D-glucopyranosyl)-cleroda-3,13(16),14-trien-17,12-olid-18-oic acid methyl ester	Aerial parts					(Choudhary et al. 2010b)
		Stems					(Parveen et al. 2019)
16	Borapetol B	Vines					(Lam et al. 2012)
		Vines	In vivo	10 µg/100 g body weight	Regulated plasma glucose levels by increasing insulin secretion	Wistar (W) and spontaneously type 2 diabetic Goto-Kakizaki (GK) rats	(Lokman et al. 2013)
17	Borapetoside B	Aerial parts	In vitro	1–100 µM	No visible cytotoxicity	MTT assay on PC-3 and 3T3 cells	(Choudhary et al. 2010b)
		Stems					(Parveen et al. 2019)
		Stems	In vivo		No inotropic effect on the electrical field-stimulated left atrium	Female Wistar rats	(Praman et al. 2013)
		Stems	In vivo	1–10 mg/kg	No effect on the blood pressure and heart rate in rats	Female Wistar rats	(Praman et al. 2012)
		Stems	In vivo	500 mg/kg	No apparent hepatotoxicity	Male ND-4 mice	(Parveen et al. 2020)
		Vines	In vivo	5 mg/kg	No significant hypoglycemic activity	Streptozotocin (STZ)-induced type-1 diabetic mice (T1DM) in ICR mice	(Lam et al. 2012; Gao et al. 2016)
18	2-*O*-lactoylborapetoside B	Vines					(Lam et al. 2012)
19	6′-*O*-lactoylborapetoside B	Vines					(Lam et al. 2012)
20	Tinosporoside A	Vines					(Gao et al. 2016)
21	(2R,5R,6R,8S,9S,10S,12S)-15,16-Epoxy-2-hydroxy-6-*O*-{β-D-glucopyranosyl-(1 → 6)-α-D-xylopyranosyl}-cleroda-3,13(16),14-trien-17,12-olid-18-oic acid methyl ester	Aerial parts					(Choudhary et al. 2010b)
22	Tinosporaside	Aerial parts					(Ahmed et al. 2006)
23	(5R,6R,8S,9R,10R,12S)-15,16-Epoxy-2-oxo-6-*O*-(β-D-glucopyranosyl)-cleroda-3,13(16),14-trien-17,12-olid-18-oic acid methyl ester	Aerial parts					(Choudhary et al. 2010b)
24	(5R,6R,8S,9R,10S,12S)-15,16-Epoxy-2-oxo-6-*O*-(β-D-glucopyranosyl)-cleroda-3,13(16),14-trien-17,12-olid-18-oic acid methyl ester	Aerial parts					(Choudhary et al. 2010b)
25	Borapetoside F	Aerial parts					(Choudhary et al. 2010b)
		Stems					(Langrand et al. 2014; Parveen et al. 2019)
		Stems	In vivo	500 mg/kg	No apparent hepatotoxicity	Male ND-4 mice	(Parveen et al. 2020)
		Vines					(Lam et al. 2012; Gao et al. 2016)
26	(2R,5R,6S,9S,10S,12S)-15,16-Epoxy-2-hydroxy-6-*O*-(β-D-glucopyranosyl)-cleroda-3,7,13(16),14-tetraen-17,12-olid-18-oic acid methyl ester (Dehydroborapetoside B)	Aerial parts					(Choudhary et al. 2010b)
		Stems					(Parveen et al. 2019)
		Vines					(Gao et al. 2016)
27	(5R,6S,9S,10S,12S)-15,16-Epoxy-2-oxo-6-*O*-(β-D-glucopyranosyl)- cleroda-3,7,13(16),14-tetraen-17,12-olid-18-oic acid methyl ester	Aerial parts					(Choudhary et al. 2010b)
28	Tinocrispol A	Vines					(Lam et al. 2012)
29	Tinosporol B	Vines					(Gao et al. 2016)
30	(3R,4R,5R,6S,8R,9S,10S,12S)-15,16-Epoxy-3,4-epoxy-6-*O*-(β-D-glucopyranosyl)-cleroda-3,13(16),14-trien-17,12-olid-18-oic acid methyl ester	Aerial parts					(Choudhary et al. 2010b)
31	Borapetol A	Stems					(Parveen et al. 2019)
		Vines					(Lam et al. 2012)
32	Borapetoside A	Aerial parts	In vitro	1–100 µM	No visible cytotoxicity	MTT assay on PC-3 and 3T3 cells	(Choudhary et al. 2010b)
		Stems	In vivo		No inotropic effect on the electrical field-stimulated left atrium	Female Wistar rats	(Praman et al. 2013)
		Stems	In vivo	1–10 mg/kg	No effect on the blood pressure and heart rate in rats	Female Wistar rats	(Praman et al. 2012)
		Vines	In vivo	5 mg/kg	Remarkably lowered serum glucose levels and reduced hepatic gluconeogenesis as well as up regulated glucose utilization	Streptozotocin (STZ)-induced type-1 diabetic mice (T1DM) in ICR mice	(Lam et al. 2012)
33	Crispene A	Stems					(Hossen et al. 2016)
34	Columbin	Aerial parts					(Ahmed et al. 2006)
		Stems					(Noman et al. 2018; Parveen et al. 2019)
		Vines					(Lam et al. 2012)
35	(1R,4S,5R,8S,9R,10S,12S)-15,16-Epoxy-4-*O*-(β-D-glucopyranosyl)- cleroda-2,13(16),14-triene-17(12),18(1)-diolide	Aerial parts					(Choudhary et al. 2010b)
36	Methyl (2R,7S,8S)-8-[(2S)-2-(3,4-dihydroxy-2,5-dimethoxytetrahydro-3-furanyl)-2-hydroxyethyl]-2,8-dimethyl-10-oxo-11-oxatricyclo[7.2.1.0^2,7^]dodec-3-ene-3-carboxylate (Rumphiol E)	Aerial parts					(Choudhary et al. 2010b)
37	Tinocrispide	Stems					(Parveen et al. 2019)
38	Baenzigeride A	Stems					(Parveen et al. 2019)
*Alkaloids*							
39	*N*-Formylanonaine	Stems					(Pachaly et al. 1992; Choudhary et al. 2010a; Hamid 2013; Ahmad et al. 2018)
		Vines	In vitro	62.5 to 1000 µg/mL	No AChE inhibitory activity	Ellman’s method	(Yusoff et al. 2014)
		Vines	In vitro		Remarkable inhibition of α-glucosidase, but not α-amylase, with respective IC_50_ values of 0.653 and 1.141 mg/mL	Enzyme inhibitory assay	(Hamid et al. 2015)
40	*N*-acetylanonaine	Stems					(Pachaly et al. 1992; Lin 2009)
41	*N*-formyldehydroanonaine	Stems					(Choudhary et al. 2010a)
42	Nuciferine	Stems					(Pachaly et al. 1992)
43	*N*-formylnornuciferine	Stems					(Pachaly et al. 1992; Bakhari et al. 2005, 2013; Choudhary et al. 2010a; Hamid 2013; Ahmad et al. 2018)
		Stems	In vitro		Significant cardiotonic activity through increasing the force of contraction on the atria of an isolated rat heart with no significant change in the heart rate		(Sunthikawinsakul 2005; Imphanban et al. 2009)
		Vines	In vitro	62.5 to 1000 µg/mL	No AChE inhibitory activity	Ellman’s method	(Yusoff et al. 2014)
		Vines	In vitro		Insignificant inhibition of α-glucosidase and α-amylase, with respective IC_50_ values of 2.409 and 1.459 mg/mL	Enzyme inhibitory assay	(Hamid et al. 2015)
44	*N*-formylasimilobine 2’-*O*-β-D-glucopyranoside	Stems					(Choudhary et al. 2010a)
45	*N*-formylasimilobine 2’-*O*-β-D-glucopyranosyl-(1 → 2)-β-D-glucopyranoside	Stems					(Choudhary et al. 2010a)
46	*N*-acetylnornuciferine	Stems					(Pachaly et al. 1992; Bakhari et al. 2005, 2013; Lin 2009)
47	*N*-demethyl-*N*-formyldehydronornuciferine	Stems					(Choudhary et al. 2010a)
48	Boldine	Stems					(Abood et al. 2014)
49	Liriodenine	Vines	In vitro		Remarkable inhibition of α-glucosidase (IC_50_ value of 0.562 mg/mL)	Enzyme inhibitory assay	(Hamid et al. 2015)
50	Lysicamine	Stems					(Bakhari et al. 2005, 2013; Hamid 2013; Ahmad et al. 2018; Parveen et al. 2019)
		Vines	In vitro		Inhibition of α-glucosidase stronger than that of α-amylase (IC_50_ values of 0.562 and 1.988 mg/mL, respectively)	Enzyme inhibitory assay	(Hamid et al. 2015)
51	Magnoflorine	Stems	In vitro		Prominent immunomodulatory activity via augmenting chemotaxis, phagocytic activity, ROS and NO productions and the secretion of IL-1β, TNF-α, IL6, PGE2 and MCP-1	RAW 264.7 macrophages	(Ahmad et al. 2018)
		Stems	In vitro		Remarkable immunomodulatory effect through upregulating various immune inflammatory-related parameters	ELISA, qRT-PCR	(Haque et al. 2018, 2020)
		Vines	In vitro	62.5 to 1000 µg/mL	No AChE inhibitory activity	Ellman’s method	(Yusoff et al. 2014)
		Vines	In vitro		Inhibition of α-amylase stronger than that of α-glucosidase (IC_50_ values of 0.957 and 2.233 mg/mL, respectively)	Enzyme inhibitory assay	(Hamid et al. 2015)
52	Berberine	Stems and Leaves					(Syarifah et al. 2017)
53	Dihydrodiscretamine	Aerial parts	In vitro		Significant antioxidant activity	DPPH free radical scavenging asssay	(Hamid et al. 2021)
		Vines	In vitro	62.5 to 1000 µg/mL	No AChE inhibitory activity	Ellman’s method	(Yusoff et al. 2014)
		Vines	In vitro		Inhibition of α-amylase stronger than that of α-glucosidase (IC_50_ values of 0.987 and 2.233 mg/mL, respectively)	Enzyme inhibitory assay	(Hamid et al. 2015)
54	Columbamine	Aerial parts	In vitro		Significant antioxidant activity	DPPH free radical scavenging asssay	(Hamid et al. 2021)
		Vines	In vitro	62.5 to 1000 µg/mL	Prominent AChE inhibitory activity with IC_50_ 48.1 µM	Ellman’s method	(Yusoff et al. 2014)
		Vines	In vitro		Weak inhibition of α-glucosidase and α-amylase (IC_50_ values of 2.934 and 1.636 mg/mL, respectively)	Enzyme inhibitory assay	(Hamid et al. 2015)
55	4,13-dihydroxy-2,8,9-trimethoxydibenzo[a,g]quinolizinium	Aerial parts	In vitro		Strong antioxidant activity	DPPH free radical scavenging asssay	(Hamid et al. 2021)
		Vines					(Yusoff et al. 2014)
56	8-methoxy palmatine	Stems					(Rahman et al. 2020)
57	( −)-Steponine	Stems					(Parveen et al. 2019)
58	Litcubinine	Stems					(Praman et al. 2011)
		Stems	In vivo		No inotropic effect on the electrical field-stimulated left atrium	Female Wistar rats	(Praman et al. 2013)
		Stems	In vivo	1–10 mg/kg	No effect on the blood pressure and heart rate	Female Wistar rats	(Praman et al. 2012)
59	Higenamine	Stems	In vivo	10^−8^–10^−5^ M	Positive inotropic effects on the isolated left atrium	Female Wistar rats	(Praman et al. 2013)
		Stems	In vivo	0.001–0.3 mg/kg	Decreased mean arterial blood pressure (MAP) and increased heart rate	Female Wistar rats	(Praman et al. 2012)
60	Paprazine (*p*-Coumaroyltyramine)	Stems					(Choudhary et al. 2010a)
61	*N*-*trans*-caffeoyltyramine	Stems					(Lin 2009)
62	*N*-*trans*-feruloyltyramine (Moupinamide)	Stems					(Choudhary et al. 2010a; Hamid 2013; Langrand et al. 2014; Noman et al. 2018; Parveen et al. 2019)
		Stems	In vitro	10 µg	Antioxidant and radical scavenging properties	DPPH assay and bleaching experiment	(Cavin et al. 1998)
		Vines	In vitro	62.5 to 1000 µg/mL	No AChE inhibitory activity	Ellman’s method	(Yusoff et al. 2014)
		Vines	In vitro		Remarkable inhibition of both α-glucosidase and α-amylase (IC_50_ values of 0.818 and 0.852 mg/mL, respectively)	Enzyme inhibitory assay	(Hamid et al. 2015)
63	*N*-*cis*-feruloyltyramine	Stems					(Langrand et al. 2014)
		Stems	In vitro	10 µg	Antioxidant and radical scavenging properties	DPPH assay and bleaching experiment	(Cavin et al. 1998)
64	Imidazolidin-4-one, 2-imino-1-(4-methoxy-6-dimethylamino-1,3,5-triazin-2-yl)	Aerial parts					(Rakib et al. 2020c)
65	Salsolinol	Stems	In vivo	10^−7^–10^−4^ M	Positive inotropic effects on the isolated left atrium	Female Wistar rats	(Praman et al. 2013)
		Stems	In vivo	0.1–10 mg/kg	Decreased MAP and heart rate in a dose-dependent manner	Female Wistar rats	(Praman et al. 2012)
66	Benzeneethanamine/ Phenethylamine	Aerial parts					(Rakib et al. 2020c)
67	Tyramine	Stems	In vivo	10^−8^–3 × 10^−5^ M	Positive inotropic effects on the isolated left atrium	Female Wistar rats	(Praman et al. 2013)
		Stems	In vivo	0.003–1 mg/kg	Increased MAP and heart rate in a dose-dependent manner	Female Wistar rats	(Praman et al. 2012)
*Flavonoids*							
68	Apigenin	Aerial parts					(Ismail and Choudhary 2016)
		Leaves	In vitro		Moderate inhibition of α-glucosidase (IC_50_ value of 34.6 µg/mL)	Enzyme inhibitory assay	(Chang et al. 2015)
69	Apigenin-7-*O*-β-D-glucoside (Cosmosiin)	Leaves	In vitro	10 µg/mL	No inhibition of α-glucosidase activity	Enzyme inhibitory assay	(Chang et al. 2015)
70	Isovitexin	Leaves	In vitro		Weak inhibition of α-glucosidase (IC_50_ value of 61.2 µg/mL)	Enzyme inhibitory assay	(Chang et al. 2015)
71	Genkwanin	Aerial parts					(Umi Kalsom and Noor 1995)
72	Genkwanin-7-*O*-β-D-glucoside	Aerial parts					(Umi Kalsom and Noor 1995)
73	Luteolin	Aerial parts					(Amom et al. 2009)
		Leaves	In vitro		Weak inhibition of α-glucosidase (IC_50_ value of 86.1 µg/mL)	Enzyme inhibitory assay	(Chang et al. 2015)
74	3'-*O*-methylluteolin	Leaves					(Chang et al. 2015)
75	Luteolin-7-*O*-β-D-glucoside	Leaves	In vitro	10 µg/mL	No inhibition of α-glucosidase activity	Enzyme inhibitory assay	(Chang et al. 2015)
76	Luteolin-4'-*O*-β-D-glucoside	Leaves	In vitro	10 µg/mL	No inhibition of α-glucosidase activity	Enzyme inhibitory assay	(Chang et al. 2015)
77	Diosmetin	Aerial parts					(Umi Kalsom and Noor 1995)
78	Luteolin-4′-methylether-7-*O*-β-D-glucoside	Aerial parts					(Umi Kalsom and Noor 1995)
79	Orientin	Leaves					(Chang et al. 2015)
80	Isoorientin	Leaves	In vitro		Insignificant inhibition of α-glucosidase (IC_50_ value of > 100 µg/mL)	Enzyme inhibitory assay	(Chang et al. 2015)
81	Morin	Aerial parts					(Amom et al. 2009)
82	Quercetin	Stems					(Abood et al. 2014)
83	Rutin	Aerial parts					(Amom et al. 2009)
84	Cosmosiin-6''-(*E*)-cinnamate	Leaves	In vitro		Remarkable inhibition of α-glucosidase activity (IC_50_ value of 11.3 µg/mL)	Enzyme inhibitory assay	(Chang et al. 2015)
85	Cosmosiin-6''-(*E*)-*p*-coumarate	Leaves	In vitro		Remarkable inhibition of α-glucosidase activity (IC_50_ value of 14.6 µg/mL)	Enzyme inhibitory assay	(Chang et al. 2015)
86	Cosmosiin-6''-(*E*)-ferulate	Leaves	In vitro		Remarkable inhibition of α-glucosidase activity (IC_50_ value of 8.8 µg/mL)	Enzyme inhibitory assay	(Chang et al. 2015)
87	Luteolin-7-*O*-β-glucosyl-6''-(*E*)-*p*-cinnamate	Leaves					(Chang et al. 2015)
88	Cosmosiin-6''-(*Z*)-*p*-coumarate	Leaves	In vitro		Remarkable inhibition of α-glucosidase activity (IC_50_ value of 10.1 µg/mL)	Enzyme inhibitory assay	(Chang et al. 2015)
89	Isovitexin-2"-(*E*)-*p*-coumarate	Leaves	In vitro		Significant inhibition of α-glucosidase activity (IC_50_ value of 4.3 µg/mL)	Enzyme inhibitory assay	(Chang et al. 2015)
90	Isoorientin 2''-(*E*)-*p*-coumarate	Leaves	In vitro		Moderate inhibition of α-glucosidase activity (IC_50_ value of 35.7 µg/mL)	Enzyme inhibitory assay	(Chang et al. 2015)
91	Isoorientin-2"-(*E*)-sinapate	Leaves					(Chang et al. 2015)
92	Catechin	Aerial parts					(Amom et al. 2009)
*Steroidal compounds*							
93	3-Ethyl-3-hydroxy-5α-androstan-17-one	Aerial parts					(Rakib et al. 2020c)
94	Callecdysterol C	Stems					(Rahman et al. 2020)
95	26,27-Dinorergosta-5,23-dien-3β-ol	Aerial parts					(Rakib et al. 2020c)
96	26,27-Dinorergost-5-ene-3β,24-diol	Aerial parts					(Rakib et al. 2020c)
97	3β-Hydroxy-5-cholen-24-oic acid	Aerial parts					(Rakib et al. 2020c)
98	Cholesterol	Aerial parts					(Rakib et al. 2020c)
99	Cholest-5-en-3-ol, 6-methyl-, (3β)- (6-methylcholesterol)	Aerial parts					(Rakib et al. 2020c)
100	26-Hydroxycholesterol	Aerial parts					(Rakib et al. 2020c)
101	25-Hydroxycholesterol, 3-methyl ether	Aerial parts					(Rakib et al. 2020c)
102	26-Homo-25-hydroxycholesterol	Aerial parts					(Rakib et al. 2020c)
103	Lathosterol	Aerial parts					(Rakib et al. 2020c)
104	Cholestane-3,5-diol, 5-acetate, (3β,5α)	Aerial parts					(Rakib et al. 2020c)
105	14-Methyl-5α-Cholest-8-en-3-one	Aerial parts					(Rakib et al. 2020c)
106	Desmosterol	Aerial parts					(Rakib et al. 2020c)
107	Cholesta-5,22-dien-3β-ol (22-dehydrocholesterol)	Aerial parts					(Rakib et al. 2020c)
108	Ergosta-5,24(28)-dien-3β-ol (24-methylencholesterol)	Aerial parts					(Rakib et al. 2020c)
109	24(R)- methylcholesta-5-en-3β-ol (Campesterol)	Stem barks					(Musa et al. 2019)
		Aerial parts					(Rakib et al. 2020c)
110	Ergost-7-en-3-ol	Aerial parts					(Rakib et al. 2020c)
111	5,6-Dihydroergosterol	Aerial parts					(Rakib et al. 2020c)
112	β-Sitosterol	Aerial parts					(Rakib et al. 2020c)
		Stems					(Noman et al. 2018)
		Vines	In vitro		Remarkable inhibition of both α-glucosidase and α-amylase (IC_50_ values of 0.582 and 0.783 mg/mL, respectively	Enzyme inhibitory assay	(Hamid et al. 2015)
113	3-*O*-β-D-Glucopyranosyl-β –sitosterol (Daucosterol)	Aerial parts					(Ismail and Choudhary 2016)
114	Stigmastan-3,5-diene	Aerial parts					(Marlina et al. 2017)
115	Stigmasterol	Aerial parts					(Rakib et al. 2020c)
		Stems					(Lin 2009)
116	Gorgost-5-en-3β-ol (Gorgosterol)	Aerial parts					(Rakib et al. 2020c)
117	9,19-Cyclocholestan-3-ol,14-methyl-(3β,5α) (Pollinasterol)	Aerial parts					(Rakib et al. 2020c)
118	Cycloeucalenol	Aerial parts					(Ismail and Choudhary 2016)
		Stems	In vitro		Mild cardiotonic effects		(Kongkathip et al. 2002)
119	Cycloeucalenone	Stems					(Noman et al. 2018)
		Stems	In vitro		Mild cardiotonic effects		(Kongkathip et al. 2002)
120	24-Methylene-9,19-cyclolanostan-3β-ol (24-Methylenecycloartanol)	Aerial parts					(Rakib et al. 2020c)
121	9,19-Cyclolanost-23-ene-3,25-diol, 3-acetate	Aerial parts					(Rakib et al. 2020c)
122	Cholest-22-ene-21-ol, 3,5-dehydro-6-methoxy	Aerial parts					(Rakib et al. 2020c)
123	20β-hydroxyecdysone	Aerial parts					(Ahmed et al. 2006)
124	Strophanthidin	Aerial parts					(Rakib et al. 2020c)
*Triterpenes*							
125	Lupeol	Aerial parts					(Rakib et al. 2020c)
		Stems					(Noman et al. 2018)
126	Lupeol acetate	Aerial parts					(Rakib et al. 2020c)
127	Lupeol, trifluoroacetate	Aerial parts					(Rakib et al. 2020c)
128	Betulin	Aerial parts					(Rakib et al. 2020c)
129	β-amyrin	Stems					(Noman et al. 2018)
*Phenolic compounds*							
130	4-Hydroxybenzaldehyde	Vines	In vitro		Remarkable inhibition of both α-glucosidase and α-amylase (IC_50_ values of 0.557 and 0.815 mg/mL, respectively)	Enzyme inhibitory assay	(Hamid et al. 2015)
131	Vanillin	Stems					(Cavin et al. 1998)
132	Methyl 3,4-dihydroxybenzoate	Aerial parts					(Ismail and Choudhary 2016)
133	3,4-Dihydroxymandelic acid	Aerial parts					(Rakib et al. 2020c)
134	Syringin	Aerial parts					(Ismail and Choudhary 2016)
		Stems					(Cavin et al. 1998; Ahmad et al. 2018)
		Stems	In vivo		No inotropic effect on the electrical field-stimulated left atrium	Female Wistar rats	(Praman et al. 2013)
		Stems	In vivo	1–10 mg/kg	No effect on the blood pressure and heart rate	Female Wistar rats	(Praman et al. 2012)
		Stems	In vitro		Remarkable immunomodulatory effect through upregulating various immune inflammatory- related parameters	Enzyme-linked immunosorbent assay (ELISA)	(Haque et al. 2020)
135	Secoisolariciresinol	Stems	In vitro	10 µg	Antioxidant and radical scavenging properties	DPPH assay and bleaching experiment	(Cavin et al. 1998)
		Stems	In vivo	10^–7^-10^–4^ M	Positive inotropic effects on the isolated left atrium	Female Wistar rats	(Praman et al. 2013)
		Stems	In vivo	0.1–10 mg/kg	Decreased MAP and heart rate (HR) in a dose-dependent manner	Female Wistar rats	(Praman et al. 2012)
136	*n*-tetracosyl trans-ferulate	Stems					(Bakhari et al. 2013)
137	( −)-Pinoresinol	Stems					(Parveen et al. 2019)
138	Syringaresinol	Aerial parts					(Chung 2011)
139	Yangambin	Aerial parts					(Rakib et al. 2020c)
*Nucleosides*							
140	Adenosine	Stems	In vivo	10^–8^ -3 × 10^–4^ M	Negative inotropic effects on the isolated left atrium	Female Wistar rats	(Praman et al. 2013)
		Stems	In vivo	0.003–0.3 mg/kg	Decreased both MAP and heart rate in a dose-dependent manner	Female Wistar rats	(Praman et al. 2012)
141	Cytidine	Aerial parts					(Choudhary et al. 2010a)
142	Uridine	Stems	In vivo	10 − 8 -10 − 2 M	Positive inotropic effects on the isolated left atrium	Female Wistar rats	(Praman et al. 2013)
		Stems	In vivo	0.1–100 mg/kg	Increased MAP and decreased heart rate	Female Wistar rats	(Praman et al. 2012)
*Aromatic compounds*							
143	Ar-Tumerone	Aerial parts					(Rakib et al. 2020c)
144	1,2-Benzenedicarboxylic acid	Stems					(Nor Aziyah et al. 2014)
145	Dibutyl phthalate	Aerial parts					(Rakib et al. 2020c)
*Monoterpenes*							
146	Camphenol	Aerial parts					(Rakib et al. 2020c)
147	Spiro[4,5]dec-6-en-1-ol, 2,6,10,10-tetramethyl	Aerial parts					(Rakib et al. 2020c)
148	(6S, 9R)-vomifoliol	Stems					(Parveen et al. 2019)
*Sesquiterpenes*							
149	Eudesma-4(15),7-dien-1β-ol	Aerial parts					(Rakib et al. 2020c)
150	Tumerone	Aerial parts					(Rakib et al. 2020c)
151	(Z)-γ-Atlantone	Aerial parts					(Rakib et al. 2020c)
152	E-*cis*, *epi*-β-Santalol	Aerial parts					(Rakib et al. 2020c)
153	α-Santalol	Aerial parts					(Rakib et al. 2020c)
154	(−)-Globulol	Aerial parts					(Rakib et al. 2020c)
*Diterpenes*							
155	*Trans*-Geranylgeraniol	Aerial parts					(Rakib et al. 2020c)
156	Retinol	Aerial parts					(Rakib et al. 2020c)
157	Retinal	Aerial parts					(Rakib et al. 2020c)
*Long chain fatty compounds*							
158	D-Mannitol, 1-*O*-(16-hydroxyhexadecyl)-	Aerial parts					(Rakib et al. 2020c)
159	13-Hydroperoxy-octadeca-9,11-dienoic acid	Stems					(Lee et al. 2020)
160	Eicosenoic acid (Paullinic acid)	Stems					(Abood et al. 2014)
161	Heneicosanoic acid, methyl ester	Aerial parts					(Rakib et al. 2020c)
162	2-Propenoic acid, dodecyl ester	Stems					(Nor Aziyah et al. 2014)
163	Ethyl pentadecanoate	Stems					(Nor Aziyah et al. 2014)
164	Oxalic acid, decyl 2-ethylhexyl ester	Stems					(Nor Aziyah et al. 2014)
165	1-Tetradecanol	Stems					(Nor Aziyah et al. 2014)
166	1-Eicosanol	Stems					(Nor Aziyah et al. 2014)
167	1-Octacosanol	Aerial parts					(Rakib et al. 2020c)
		Stems					(Ahmad et al. 2018)
		Stems					(Bakhari et al. 2013)

### Clerodane-type furanoditerpenoids

Furanoditerpenoids are a class of compounds that features at least one furan ring as part of their core skeleton. The outstanding significance of this class lies in its pharmacological potential, which is primarily be attributed to the biologically-interactive furan ring. The clerodane-type furanoditerpenoids are based on a decahydronaphthalene skeleton with a furan ring attached to it via a two-carbon bridge. Based on the number of lactone rings present, these compounds have been further categorized into three major subgroups viz. A, B and C, featuring zero, one and two lactone rings, respectively (Bao et al. 2017). A total of 38 clerodane-type furanoditerpenoids have been identified in *T. crispa* (**1**–**38**) (Fig. 2). Among them, only two (**1**, **2**) were of type A (Hossen et al. 2016; Noman et al. 2018), while 28 compounds (**3**–**30**) featured one lactone ring in their structures and belonged to type B (Ruan et al. 2012; Lokman et al. 2013; Abood et al. 2014; Langrand et al. 2014; Hamid et al. 2015; Mantaj et al. 2015; Adnan et al. 2016; Gao et al. 2016; Xu et al. 2017; Rahman et al. 2020). Five of the furanoditerpenoids (**31**–**35**) were of type C with two lactone rings (Ahmed et al. 2006; Choudhary et al. 2010b; Lam et al. 2012; Praman et al. 2012). Compounds from both type B and C exhibited further structural diversification in terms of the position of the lactone ring(s), extent of hydroxylation and presence of monosaccharides at different positions. A total of 21 furanoditerpenoids (**4**–**6**, **8**, **13**–**15**, **17**–**27**, **30**, **32**, **35**) were characterized as glycosides. While most of the glycosidic constituents contained a single β-D-glucose moiety in their structure, two of them (**6**, **21**) featured two saccharide moieties (Gao et al. 2016), and one of them (**21**) included an α-D-xylose moiety (Choudhary et al. 2010b). The furanoditerpenoids isolated from *T. crispa* also included three re-arranged derivatives, including compound (**36**) with a saturated furan ring and extensive hydroxylation on all side chains (Choudhary et al. 2010b) and compounds (**37**, **38**) with a shortened first ring in the basic skeleton along with a fusion of a five-membered lactone ring (Parveen et al. 2019).

### Alkaloids

Alkaloids reported from *T. crispa* mostly originated from the structural extension of the basic isoquinoline skeleton. Thirteen aporphine alkaloids (**39**–**51**) have been isolated from different parts of *T. crispa* (Fig. 3) (Pachaly et al. 1992; Bakhari et al. 2005, 2013; Sunthikawinsakul 2005; Imphanban et al. 2009; Choudhary et al. 2010a; Hamid 2013; Yusoff et al. 2014; Hamid et al. 2015; Ahmad et al. 2018; Parveen et al. 2019). Five protoberberine-type alkaloids (**52**–**56**) have also been reported (Yusoff et al. 2014; Hamid et al. 2015, 2021; Syarifah et al. 2017; Rahman et al. 2020). Both aporphine and protoberberine alkaloids feature a tetracyclic skeleton based on the benzylisoquinoline moiety, originating from the oxidative fusion of phenol and isoquinoline rings, with partial or complete aromatization. However, these alkaloids differ in the orientation of their fusion. The bridging in aporphine-based structures takes place along the middle of the isoquinoline skeleton without incorporating the nitrogen atom into the extended ring (Ge and Wang 2018). On the other hand, in protoberberine alkaloids, the incoming phenol fuses along the *N*-methyl group and incorporates nitrogen into the new ring (Da-Cunha et al. 2005). Two similarly-fused isoquinoline alkaloids (**57**, **58**) and one simple isoquinoline alkaloid (**59**) have also been isolated from the stems of *T. crispa* (Praman et al. 2011, 2012, 2013; Parveen et al. 2019). Eight other alkaloids (**60**–**67**), including four hydroxycinnamoyl tyramine derivatives (**60**–**63**) along with tyramine itself (**67**), have also been reported (Cavin et al. 1998; Choudhary et al. 2010a; Praman et al. 2012, 2013; Hamid 2013; Langrand et al. 2014; Yusoff et al. 2014; Noman et al. 2018; Parveen et al. 2019; Rakib et al. 2020c).

### Flavonoids

Different parts of *T. crispa* have been characterized with the presence of 24 flavones (**68**–**91**) and one flavanol (**92**) (Fig. 4) (Umi Kalsom and Noor 1995; Amom et al. 2009; Abood et al. 2014; Chang et al. 2015). Among the flavones, 16 compounds (**69**, **70**, **72**, **75**, **76**, **78**–**80**, **84**–**91**) were identified as glucosides while (**83**) was identified as a rutinoside. Eight of these flavones (**84**–**91**) were further conjugated with hydroxycinnamoyl moieties.

### Steroidal compounds

Thirty-two steroidal constituents (**93**–**124**) have been isolated from *T. crispa* (Fig. 5) (Ahmed et al. 2006; Lin 2009; Hamid et al. 2015; Ismail and Choudhary 2016; Marlina et al. 2017; Noman et al. 2018; Musa et al. 2019; Rahman et al. 2020; Rakib et al. 2020c). All compounds displayed the characteristic steroidal backbone and showed diversity in their unsaturation, oxidation and cyclization in different parts of this backbone.

### Triterpenes

Four lupane-based (**125**–**128**) and one oleanane-based (**129**) pentacyclic triterpenes have been isolated from the aerial parts and stems of *T. crispa* (Fig. 6) (Noman et al. 2018; Rakib et al. 2020c).

### Phenolic compounds

Ten phenolic constituents (**130**–**139**) have been identified in *T. crispa* (Fig. 7), including one (**134**) identified as a glucoside (Cavin et al. 1998; Praman et al. 2012, 2013; Hamid et al. 2015; Ismail and Choudhary 2016; Ahmad et al. 2018; Rakib et al. 2020c). One of the phenolics (**136**) was the ester product of a hydroxycinnamoyl derivative (Bakhari et al. 2013), whereas three of them (**137**–**139**) were polyphenolic lignans (Chung 2011; Parveen et al. 2019; Rakib et al. 2020c). Although hydroxycinnamoyl conjugations are common within the alkaloidal and flavonoid pool of *T. crispa*, the presence of hydroxycinnamic acids has never been reported and warrants future attention.

### Other constituents

Less prominent secondary metabolites, including three nucleosides (**140**–**142**) (Choudhary et al. 2010a; Praman et al. 2012, 2013), three aromatic compounds (**143**–**145**) (Nor Aziyah et al. 2014; Rakib et al. 2020c), three volatile monoterpenes (**146**–**148**), six volatile sesquiterpenes (**149**–**154**), three volatile diterpenes (**155**–**157**) (Rakib et al. 2020c) and ten long chain alcohols and fatty acid derivatives (**158**–**167**) (Fig. 8) (Bakhari et al. 2013; Abood et al. 2014; Nor Aziyah et al. 2014; Ahmad et al. 2018; Lee et al. 2020; Rakib et al. 2020c) have also been reported in *T. crispa*.

## Pharmacological activity

*Tinospora crispa* has been extensively studied in vitro*, *in vivo and in silico to scientifically validate its use in ethnomedicine. Most studies have focussed on the antidiabetic and cardiac activity, including the mechanisms of action at the molecular level, of *T. crispa* extracts and phytoconstituents. Significant evidence to support the anticancer, antiparasitic, antimicrobial, antioxidant and immunomodulatory potential of this plant has also been obtained. Preliminary evidence of its hepatoprotective, analgesic, antipyretic, anticholinesterase, central nervous system, antihyperuricemic and pesticidal activity has been reported. Such effects, however, remain comparatively unexplored and require further exhaustive investigations. A concise summary of the pharmacological activities of the plant is presented in Table 3.

**Table 3:** Pharmacological activities of *Tinospora crispa*

Activity	Preparation type	Study type	Testing subjects/ methods	Dose administered	Effects	References
Antidiabetic	Aqueous extract	In vivo	Male Wistar albino rats	4 mg/mL	Increased insulin secretion and lowered serum glucose levels	(Noor et al. 1989)
		In vitro	Rat islets of Langerhans	0.01–1 mg/mL	Raised both basal and glucose-induced insulin concentration	
		In vitro	Human islets of Langerhans	1 mg/mL	Raised both basal and glucose-induced insulin concentration	
		In vitro	HIT-T15 cells	0.01–4.00 mg/mL	Raised both basal and glucose- induced insulin concentration	
	Aqueous extract	In vitro	HIT-T15 cells	1 mg/mL	Showed antidiabetic effect through changing of calcium ion concentration in β-cell	(Noor and Ashcroft 1998a)
	*T. crispa* powder (capsule)	In vivo	Type-2 diabetes patients	1 g thrice daily	Confirmed antidiabetic property only via insulinotropic action	(Sangsuwan et al. 2004)
	Aqueous extract	In vitro	L6 myotubes	100–1000 µg/mL	Anti-hyperglycemic action by increasing glucose uptake, secretion of AMPK and mRNA levels of Glucose Transporter 1 (GLUT1)	(Noipha et al. 2011)
	Aqueous extract	In vivo	Wistar Rats	1 g/mL	Mainly lowered serum glucose concentration. Also curtailed cholesterol, triglycerides, aspartate transaminase, alanine transaminase, total protein, creatine and urea levels	(Abu et al. 2015)
	Methanol extract of stem	In vitro	Insulin resistant Hep-G2 cells	100 µg/mL	Enhanced glucose uptake via increasing expression of insulin receptor and GLUT4	(Abu et al. 2017)
	Ethanol extract of stem	In vitro	α-Glucosidase enzyme	450 ppm	α-Glucosidase inhibitory activity	(Tambunan et al. 2013)
	Aqueous extract	In vivo	Diabetic Sprague Dawley rats	500 mg/kg	Upregulated Superoxide Dismutase (SOD) and Glutathione Peroxidase (GPx) levels	(Firdausa et al. 2018)
	Ethanol and aqueous extracts of the stem	In vitro	α-Amylase enzyme	4–20 mg/mL	α-Amylase inhibitory activity (IC_50_ of 10.348 ± 0.313 and 11.660 ± 0.310 mg/mL, respectively)	(Hartini et al. 2022)
Cardiac	Petroleum ether, chloroform, methanol, aqueous extracts and four fractions from the chloroform extract of stems	In vitro	Isolated atria and aorta of male Sprague Dawley rats	0.25–1 mg/mL	Potent cardiac activity via non-competitive α and β adrenoceptor antagonists as well as abated isoprenaline induced positive chronotropic response	(Bakhari and Isa 2010)
	*n*-Butanol fraction of aqueous extract	In vivo	Female Wistar rats	1–100 mg/kg	Remarkable hypotensive and positive chronotropic action	(Praman et al. 2011)
Anticancer	Petroleum ether fraction of the methanol extract	In vitro	Brine shrimp lethality assay		Prominent cytotoxicity (IC_50_ value of 173 ppm)	(Mackeen et al. 2000)
	Methanol extract, chloroform and petroleum ether fractions of the stem	In vitro	Brine shrimp lethality assay	0.781–400 μg/mL	Potent anticancer activity	(Haque et al. 2011)
	Methanol extract, chloroform and petroleum ether fractions of the stem	In vitro	Brine shrimp lethality assay	0.781–400 μg/mL	Significant cytotoxic potential	(Islam et al. 2013)
	Ethanol extract of leaves	In vitro	Brine shrimp lethality assay	10–1000 μg/mL	Moderate cytotoxicity	(Tarukbua et al. 2018)
	Methanol extract	In vitro	HL-60, HEP-G2 and Hep3B cancer cells	-	Dose and time-dependent suppression of proliferation	(Ahmad et al. 2016a)
	Aqueous extract	In vitro	MCF-7, Caov-3, HeLa and HEP-G2 cells		Moderate anti-proliferative activity (IC_50_ value of 107, 100, 165 and 165 μg/mL, respectively)	(Zulkhairi Jr et al. 2008)
	Aqueous, methanol and chloroform extracts of stems	In vitro	MCF-7, MDA-MB-231, 3T3 and HeLa cells/ MTT assay	10 -100 μg/mL	Dose-dependent cytotoxicity (maximum potency observed for the aqueous extract)	(Ibahim et al. 2011)
	Ethanol extract	In vitro	HN22 and HSC3 cell lines/ MTT assay, RT-PCR, ELISA	12.5, 25, 50, and 100 μg/mL	Inhibited cancer development at the metastasis stage. Attenuated MMP-13 gene expression. At higher doses, enhanced TIMP-2 levels in HSC-3 cells	(Phienwej et al. 2015)
	Chloroform extract of the stem	In vitro	Chick embryo / Chorioallantoic Membrane (CAM) method	15, 60, 240, and 960 μg/mL	Prominent anti-angiogenic action in a dose-dependent manner	(Triastuti 2010)
	Methanol and aqueous extracts of the stem	In vitro	HL-60, HEP-G2 and MCF-7 cancer cells/ WST and MTT assay	5–500 μg/mL	Negligible cytotoxic potential	(Tungpradit et al. 2010)
	Ethanol extract	In vitro	MCF-7 breast cancer cells/MTT assay	7–16%	Variable cytotoxicity with LC_50_ values from 30.64 ± 2.18 to 254.15 ± 30.77 μg /mL	(Mutiah et al. 2019)
Antiparasitic	Methanol extract of the stem	In vitro	*Plasmodium falciparum* (FCR-3 strain)	0.1–2.5 mg/mL	Prominent antimalarial activity	(Rahman et al. 1999)
		In vivo	Adult female ddy mice, infected with *P. berghei* (ANKA strain)	5 mg/kg	Mild activity	
	Methanol extract of the stem	In vivo	*P. berghei* (ANKA strain)	20, 100, 200 mg/kg	Mild activity	(Niljan et al. 2014)
	Aqueous extract	In vitro	*P. falciparum* and *Babesia gibsoni*	1 mg/mL	Significant activity against *B. gibsoni*	(Murnigsih et al. 2005)
	Ethanol, ethyl acetate and *n*-hexane fractions of the stem	In vitro	*P. falciparum*	0–50 μg/mL	Poorly active	(Ramadani et al. 2018)
	Methanol extract	In vitro	*P. falciparum* (3D7 strain) / Percent parasitemia assay	0.5–3.0 mg/mL	Effectively reduced percent parasitemia and amount of parasite DNA dose-dependently up to a concentration of 2 mg/mL	(Ihwan et al. 2014)
	Ethanol extract	In vitro	*P. falciparum* (3D7 strain) / Percent parasitemia assay		Strong activity (IC_50_ value 0.344 ± 0.210 µg/mL)	(Abdillah et al. 2015)
		In vivo	Male Swiss mice infected with P*. berghei* (NK65 strain)	50–400 mg/kg	Moderate activity	
	Methanol extract	In vitro	*P. falciparum* FCR-3		Prominent antimalarial effect (EC_50_ value 7.5 µg/mL)	(Tran et al. 2003)
	Ethanol extract	In vivo	ICR mice infected with *P. yoelii* (17XLstrain) / Percent parasitemia assay	20, 40 and 80 mg/kg	Effective antimalarial activity in a dose-dependent manner	(Rungruang and Boonmars 2009)
	Aqueous extract	In vivo	ICR mice infected with *P. berghei*	500 mg/kg	Hepatoprotective action through attenuating the concentration of serum alanine aminotransferase (ALT) and aspartate aminotransferase (AST)	(Somsak et al. 2015)
	Aqueous extract	In vivo	ICR mice infected with *P. berghei*	500, 1000 and 2000 mg/kg	Demonstrated renoprotective and anti-hemolytic effects	(Nutham et al. 2015)
	Aqueous extract	In vivo	C57BL/6 J mice infected with *P. berghei*	2.5, 3 and 3.5 mg/kg in combination with artesunate (32 mg/kg)	Diminished the concentration of Nuclear Factor Kappa B (NFκB) and Intracellular Adhesion Molecule- 1 (ICAM1)	(Izzati et al. 2016)
	Aqueous extract of stem	In vitro	*Brugia malayi*/antifilarial assay	1, 5 and 10 mg/mL	Reduced mobility discernibly	(Zaridah et al. 2001)
	Oil extract of the stem used as an ointment	In vitro	*Pediculus humanus capitis*	15 mg	Remarkable pediculicidal activity	(Torre et al. 2017)
	Ethanol extract of the stem	In vitro	Vero cell line/ MTT assay	1.56–200 μg/mL	Relatively active against *Toxoplasma gondii* (RH strain)	(Sharif et al. 2019)
		In vitro	Anti-toxoplasma assay	1.56–200 μg/mL	Potent anti-toxoplasma potential was noted	
	*n*-hexane, chloroform, methanol, and distilled water extracts	In vivo	*Pomacea canaliculata*	1000, 5000 and 10,000 ppm	Molluscidal activity with LC_50_ of 14.771, 5.888, 3.428 and 14.993 ppm, respectively, using Probit analysis	(Aziz et al. 2021)
Antimicrobial	Aqueous, ethanol and chloroform extracts	In vitro	*Staphylococcus aureus, Streptococcus pneumoniae, Corynebacterium diphtheriae, Bacillus cereus, Listeria monocytogenes, Escherichia coli, Salmonella typhi, Shigella flexneri, Klebsiella pneumoniae, Proteus vulgaris/* disk diffusion assay	25, 50, 75, and 100%	Dose-dependent inhibition against *S. pneumoniae, C. diphtheriae* and *S. flexneri*	(Zakaria et al. 2006)
					Inhibition of *S. aureus* and *E. coli* by the aqueous and chloroform extracts at concentrations above 50%	
					No activity against *B. cereus* and *S. typhi*	
	Aqueous extract	In vitro	*S. aureus* and *E. coli* / agar diffusion assay	0.89—227.27 mg/mL	Moderate activity	(Zakaria et al. 2011)
	Aqueous, ethanol, methanol and chloroform extracts	In vitro	*S. pneumoniae, E. coli* and *Candida albicans/* disk diffusion assay	100 μg/disk	Activity comparable to the standards tetracycline and fluconazole	(Asif Iqbal et al. 2012)
	Ethanol extract	In vitro	Methicillin Resistant *S. aureus (MRSA)*/ disk diffusion assay	1 mg/disk	Activity in comparison to the standard vancomycin	(Al-alusi et al. 2010)
	Ethanol extract	In vitro	*S. aureus* and *Pseudomonas aeruginosa*	9% v/v as ointment with zeolite	Bactericidal activity	(Susanti et al. 2016)
	Ethanol extract	In vitro	*E. coli*	8% and 32%	Significant inhibition of bacterial growth	(Muslimin et al. 2018)
	Ethanol extract	In vitro	*Trichophyton rubrum /* agar diffusion assay	≥ 40%	Potent antifungal activity	(Erza et al. 2020)
	*n*-hexane extract	In vitro	*S. aureus, S. boydii, S. dysenteriae, V. mimicus, C. albicans and A. niger* / disc diffusion assay	400 μg/disc	Pronounced zones of inhibition observed	(Rahman et al. 2020)
	Plant ethanol extract	In vitro	*Propionibacterium acnes*	30% ointment	Anti-acne activity	(Yusriani et al. 2018)
	Chloroform fraction of the methanol extract	In vitro	*B. subtilis, B. megaterium, S. aureus, Sarcina lutea, E. coli, Shigella dysenteriae, S. typhi, S. paratyphi, Shigella boydii, Vibrio mimicus, V. parahemolyticus, C. albicans, A. niger* and *Sacharomyces cerevisiae/* disk diffusion assay	400 μg/disc	Mild inhibition of bacterial growth	(Haque et al. 2011)
	Chloroform fraction of the methanol extract	In vitro	*B. subtilis, B. megaterium, S. aureus, P. aeruginosa, S. lutea, E. coli, S. dysenteriae, S. typhi, S. paratyphi, S. boydii, V. mimicus, V. parahemolyticus, C. albicans, A. niger* and *S. cerevisiae*	400 μg/disc	Mild inhibition of bacterial growth	(Islam et al. 2014)
	Protein extract	In vitro	*B. cereus, S. aureus, K. pneumonia* and *Salmonella typhimurium* / disc diffusion assay	-	Inhibition of *B. cereus* growth	(Zin et al. 2016)
	Ethanol and aqueous extracts	In vitro	HIV-1 integrase	3–100 µg/mL	Mild activity (IC_50_ > 100 µg/mL)	(Bunluepuech and Tewtrakul 2009)
Immunomodulatory	Methanol extract of the stem	In vivo	Rats	10 mg/kg	Suppression of edema development	(Higashino et al. 1992)
	*n*-Butanol fraction	In vivo	Rats	3 mg/kg	Prominent activity	
	Ethanol and aqueous extract of the stem	In vitro	Rat basophilic leukemia (RBL)- 2H3 cells	0–100 μg/mL	Weak anti-inflammatory activity	(Kraithep et al. 2008)
	Methanol extract	In vitro	Luminol/lucigenin based chemiluminescence assay	0.78, 1.56, 3.13, 6.25, 12.5 μg/mL	Effective reduction of ROS levels	(Jantan et al. 2011)
	Methanol extract	In vitro	PMN chemotaxis assay	0.625, 1.25, 2.5, 5, 10 μg/mL	Mild activity	
	Methanol and aqueous extracts of the stem	In vitro	Human umbilical vein endothelial (HUVEC) cells	100–600 μg/mL	Reduced secretion of Intracellular Adhesion Molecule- 1 (ICAM-1), Vascular Cell Adhesion Molecule-1 (VCAM-1), and increased NO levels	(Kamarazaman et al. 2012)
	Aqueous extract	In vivo	Albino rats	50, 100 and 150 mg/kg	Reduction of edema development	(Hipol et al. 2012)
		In vitro	Human RBC and albumin	5 and 7.5%	Noticeable membrane stabilizing activity	
	Ethanol extract	In vivo	Male Balb/C mice	50, 100 and 200 mg/kg	Upregulation of phagocytosis, synthesis of NO, lysozyme and myeloperoxidase	(Ahmad et al. 2016b)
	Ethanol extract	In vitro	RAW 264.7 cell line/ Flow cytometry immunostaining assay	25–1000 μg/mL	Increased production of pro-inflammatory cytokines	(Abood et al. 2014)
	Ethanol extract	In vitro	RAW 264.7 cell line/ chemotaxis assay	12.5–200 μg/mL	Significant immunomodulatory activity	(Ahmad et al. 2018)
	Ethanol extract	In vitro	U397 human macrophages	0.125–75 μg/mL	Potentiated expression of NFκB, IL-1β and TNF-α; prominently increased cyclooxygenase-2 (COX-2) and PGE_2_ activity	(Haque et al. 2020)
	Freeze dried aqueous extract	In vitro	Human RBC	100–800 μg/mL	Non-hemolytic and membrane stabilizing activity	(Adnan et al. 2019)
Antioxidant	Methanol, aqueous and chloroform extracts	In vitro	DPPH free radical scavenging assay		Strong antioxidant activity (IC_50_ value of 12 μg/mL)	(Ibahim et al. 2011)
	Ethanol extract, aqueous and ethyl acetate fractions	In vitro	DPPH free radical scavenging assay	200 μg/mL	Potent antioxidant potential	(Irianti et al. 2011)
	Aqueous extract	In vitro	DPPH free radical scavenging, Thiobarbituric Acid (TBA) and Ferric Reducing Antioxidant Power (FRAP) assays	10%	Remarkable DPPH, Thiobarbituric Acid (TBA) inhibition, and Ferric Reducing Antioxidant Power (FRAP)	(Amom et al. 2011)
		In vivo	Hypercholesterolemic rabbits	150, 300 and 450 mg/kg	Antioxidant potential, inhibition of hypercholesterolemia and atherosclerosis	
	Carbon tetrachloride fraction	In vitro	DPPH free radical scavenging assay	0.98- 500 μg/mL	Strong antioxidant potential	(Haque et al. 2011)
	Methanol extract, petroleum ether, chloroform, and aqueous fractions	In vitro	DPPH free radical scavenging assay	0.98- 500 μg/mL	Moderate antioxidant activity	
	Ethanol extract, aqueous fraction and different subfractions	In vitro	DPPH free radical scavenging assay		Significant activity with respective IC_50_ values of 49.92 μg/mL, 38.25 μg/mL, 36.12 μg/mL, and 16.18 μg/mL	(Warsinah et al. 2020)
	Petroleum ether, chloroform, methanol and aqueous extracts	In vitro	Metal chelating assay	1 mg/mL	Suppression of ferrozine-Fe^2+^ complex formation	(Zulkefli et al. 2013)
	Methanol extract	In vitro	H_2_O_2_ induced HUVEC cells/ MTT cell viability assay	400, and 600 μg/mL	Increased cell viability	(Kamarazaman et al. 2012)
	Aqueous extract	In vitro	H_2_O_2_ induced HUVEC cells/ MTT cell viability assay	50–1000 μg/mL	Increased cell viability and production of several antioxidant enzymes	
	Aqueous extract	In vivo	Rabbits	200, 450 and 600 mg/kg	Enhanced SOD and GPx activity	(Zamree et al. 2015)
	Aqueous extract of stem	In vivo	Adult male New Zealand albino rabbits	200, 450 and 600 mg/kg	Decreased atherosclerotic plaque coverage, CRP levels and foam cell formation	(Shah et al. 2021)
Hepatoprotective	Methanol extract	In vivo	Male Sprague Dawley rat	0.001–1.0 mg/mL	Enhanced aminopyrine *N*-demethylase enzyme activity	(Tin et al. 2005)
	Methanol extract	In vitro	-	0.5 mg/mL	Significant inhibition of CYP3A4 enzyme	(Usia et al. 2006)
	Methanol extract	In vitro	-	1.65 mg/mL	Significant inhibition of CYP3A4 and CYP2D6 enzymes	(Subehan et al. 2006)
	Ethanol extract	In vitro	HEP-G2 cells	400 μg/mL	Hepatoprotective activity via expression of HO-1	(Lee et al. 2017)
	Methanol extract	In vivo	Swiss albino mice	100–400 mg/kg	Significant reduction of ALT, AST, Alkaline Phosphatase (AP), Malondialdehyde (MDA) and total bilirubin levels	(Rakib et al. 2020a)
Analgesic	Dried Stem	In vivo	-	-	Central analgesic activity	(Almeida et al. 2001)
	Ethanol extract	In vivo	Swiss albino mice / acetic acid-induced writhing method	300 mg/kg	Prominent analgesic activity	(Sulaiman et al. 2008)
	Methanol extract, petroleum ether and chloroform fractions	In vivo	Swiss albino mice / acetic acid-induced writhing method	400 mg/kg	Petroleum ether fraction exhibited the most significant peripheral analgesic activity compared to other fraction	(Islam et al. 2014)
	Methanol extract and chloroform fraction	In vivo	Swiss albino mice/ acetic acid-induced writhing and formalin induced paw-licking tests	200 mg/kg; 400 mg/kg	Marked anti-nociceptive activity	(Rakib et al. 2020b)
Antipyretic	*n*-butanol fraction	In vivo	Rats	3 mg/kg	Potent antipyretic activity	(Higashino et al. 1992)
	Ethanol extract	In vivo	Male Wistar rats	20- 80%	Attenuation of induced fever	(Wulandari and Bestari 2016)
	Methanol extract, petroleum ether and *n*-hexane fractions	In vivo	Swiss albino mice	400 mg/kg	Remarkable antipyretic activity	(Rakib et al. 2020a)
CNS	Decoction of the plant	In vivo	Male albino mice/ motor activity test, curiosity test, hanging test and rotary road test	6.5, 13 and 26%	CNS stimulant effect at the lowest dose	(Merwanta et al. 2019)
	Methanol extract, chloroform and *n*-hexane fractions	In vivo	Swiss albino mice/ Open Field test	200 and 400 mg/kg	Effective reduction of mobility	(Rakib et al. 2020b)
	Methanol extract, chloroform fraction	In vivo	Swiss albino mice/ Elevated Plus Maze test	200 and 400 mg/kg	Anxiolytic activity	
Antihyperuricemic	*n*-hexane insoluble fraction of ethanol extract	In vivo	Male BALB/C mice	50–200 mg/kg	Hypo-uricemic activity at doses of 50 and 100 mg/kg. Significantly greater activity recorded at 100 mg/kg compared to the standard allopurinol	(Harwoko and Warsinah 2020)
	Root extract	In vivo	Male BALB/C mice	1%	No visible activity	(Vikneswaran and Chan 2005)
Pesticidal	Ethanol and petroleum ether extracts	In vivo	*Spinacia oleracea* (Spinach plants)	-	Significant reduction of the moth (*Spodoptera exigua)* population	(Isa et al. 2013)
	Ethanol and ethyl acetate extracts	In vivo	*Brassica juncea rugosa* (Mustard plants)	1 g/L	Significant reduction of the moth(*Phyliotera sinuata ateph)* population	(Nor Aziyah et al. 2014)
	Ethanol extract	In vivo	Chinese kale leaf	0.312, 0.625, 1.25, 2.5 and 5%	Significant reduction of the moth (*Plutella xylostella*) larvae population	(Suvannarat et al. 2015)
	Petroleum ether of the mature fruit	In vitro	*Culex quinquefasciatus* larvae	(80–160 ppm)	LC_50_ ranging from 79.58–127.19 mg/L at different growth phases	(Pal et al. 2016)
	Aqueous extract of the stem	In vitro	*Culex quinquefasciatus* larvae	3.125, 6.25, 12.5, and 25 mg/L	LC_50_ and LC_90_ values of 16.95 and 30.12 mg/L	(Jiraungkoorskul 2019)

### Antidiabetic activity

The aqueous extract of *T. crispa* has been evaluated for its activity on diabetic male Wistar albino rats, on rat and human islets of Langerhans, and on HIT-T15 cells. A week after administration of the extract (4 mg/mL), lowered blood glucose levels (10.4 ± 1.0 mmol/L) were observed compared to the control group (17.4 ± 1.7 mmol/L). Additionally, insulinotropic activity was also evident with comparatively greater insulin levels in the test group than in the control (12.8 ± 1.1 µU/mL and 8.0 ± 0.7 µU/mL, respectively). In the rat islets, the extract (0.01–1 mg/mL) led to a dose-dependent enhancement of basal insulin secretion up to a maximum of fivefold. The extract also potentiated (1.5-fold) the glucose-mediated induction of basal insulin secretion. Similar results were obtained in the human islet system as the extract (1 mg/mL) induced insulin release similar to that of a high dose of glucose (20 mmol/L). The extract also further potentiated glucose-mediated insulin release. In HIT-T15 cells, the extract (0.01–4.00 mg/mL) boosted the basal insulin release sevenfold, along with a 1.5-fold enhancement of glucose-induced insulin secretion. This was the first evidence of the plant acting as an oral hypoglycemic and insulinotropic agent (Noor et al. 1989). The in vivo antidiabetic effect was further confirmed by multiple subsequent studies in other animal models (Arcueno et al. 2015; Hassani et al. 2016; Arundina et al. 2017; Firdausa et al. 2020).

Antidiabetic mechanisms other than an insulinotropic effect were evaluated in another study using the aqueous extract. It was found that the extract (1 mg/mL) played no significant role in intestinal or adipocyte glucose uptake. In HIT-T15 cells, the insulinotropic activity was inhibited by adrenaline (5 mM), somatostatin (1 mg/mL), verapamil (50 mM) and nifedipine (50 mM). Cyclic AMP concentration (cAMP) and ^86^Rb efflux were further measured and it was hypothesized that the insulinotropic effect of *T. crispa* was the result of calcium ion transport across the membrane of pancreatic β cells, and possibly closure of ATP-mediated potassium channels (Noor and Ashcroft 1998a). This was confirmed by a later study which revealed that the extract increased HIT-T15 cell sensitivity to extracellular calcium ions and resulted in increased intracellular accumulation of these ions caused by increased uptake and suppressed efflux. The physiological nature of the underlying mechanism suggested the presence of individual compounds in *T. crispa* which may serve as potential insulin secretagogues (Noor and Ashcroft 1998b). It was found in a later study that the administration of *T. crispa* powder in capsule form (1 g thrice daily) could not induce hypoglycemia in type-2 diabetic patients non-responsive to oral hypoglycemic drugs. It was postulated that these results reaffirm the insulinotropic nature of the antidiabetic activity of *T. crispa* (Sangsuwan et al. 2004).

An increase in glucose uptake and Glucose Transporter 1 (GLUT1) expression was reported when testing an aqueous extract of *T. crispa* on L6 myotubes. 2-Deoxy-[^3^H]-glucose (2-DG) uptake was measured following incubation up to 24 h with 100–1000 µg/mL of extract. At a dose of 400 µg/mL, 2-DG uptake increased by 151.5 ± 1.1, 166.7 ± 15.0, 179.6 ± 6.8 and 246.1 ± 0.1% following 4, 6, 8, and 24 h of incubation, respectively. The same dose also displayed a steady increase in mRNA levels of GLUT1 by 1.29 ± 0.06, 1.70 ± 0.22, and 2.04 ± 0.23 fold over a course of 4, 8 and 24 h, respectively. These were accompanied by boosted levels of extracellular signal-regulated kinases (ERK) 1/2, suggesting that this pathway is activated causing the increased GLUT1 expression. Increased AMPK levels were also observed in L6 myotubes (Noipha et al. 2011).

This ability to reverse the insulin resistance was also demonstrated in a study using Wistar rats fed a high fat diet. The aqueous extract of *T. crispa* at a dose of 1 g/mL resulted in a significant decrease in glucose (8.50 ± 0.30 mmol/L compared to 13.75 ± 0.25 mmol/L in the untreated group). Serum glucose, cholesterol and triglycerides levels decreased with the treatment, along with a fall in serum alanine aminotransferase (ALT), aspartate aminotransferase (AST), total protein, creatine and urea (Abu et al. 2015). A subsequent investigation established the capacity to abolish insulin resistance in insulin resistant IR-HEP-G2 cells using rosiglitazone maleate as a standard. It was observed that *T. crispa* methanol extract and the standard (both at doses of 100 µg/mL) led to a 2.5- and 1.5-fold increase in 2-DG uptake, respectively. It was found that the insulin receptor was upregulated, ultimately recruiting the PI3K/Akt pathways. Subsequent increase of GLUT4 expression was also observed resulting in a boosted 2-DG uptake. Additionally, *T. crispa* methanol extract triggered apoptosis in the IR-HEPG2 cells stimulated with insulin (Abu et al. 2017).

Another study revealed that an ethanol extract of *T. crispa* stems displayed α-glucosidase inhibitory activity, with a 78.34% inhibition at a concentration of 450 ppm compared to 81.01% when using the standard acarbose. The IC_50_ values for the extract and acarbose were 237 and 116 ppm, respectively (Tambunan et al. 2013). In a recent study, the ethanol and aqueous extracts of the stem have also been observed to inhibit the enzyme α-amylase in vitro with an IC_50_ of 10.348 ± 0.313 and 11.660 ± 0.310 mg/mL, respectively (Hartini et al. 2022). Interestingly, endophytic fungi isolated from *T. crispa* have been found to exhibit α amylase and α glucosidase inhibitory activity (Lestari et al. 2015; Pramitasari et al. 2017). The aqueous extract of the plant at a dose of 500 mg/kg has been reported to increase superoxide dismutase (SOD) and glutathione peroxidase (GPx) levels in streptozotocin-treated diabetic Sprague Dawley rats, thereby boosting antioxidant activity (Firdausa et al. 2018). The ethanol extract of *T. crispa* has showed an ability to increase lymphocytes, fibroblasts and enhanced healing activity in diabetic male Wistar rats with oral mucosal ulcers (Arundina et al. 2017; Roestamadji et al. 2017).

As there have been numerous studies on the antidiabetic potential of *T. crispa* extracts, the same can also be said for its phytoconstituents. Particularly, a number of clerodane type furanoditerpenoids and their glycosides have been reported to have significant hypoglycemic activity. Borapetosides A **(32)** and C **(14)** at a dose of 5 mg/kg significantly decreased blood glucose levels in normal and type-1 diabetic mice compared to the standard metformin (200 mg/kg). Borapetoside C **(14)** at a dose of 3 mg/kg also displayed activity against type-2 diabetes, evident from its insulin secretagogue activity. This was comparable to that of glibenclamide (5 mg/kg) and was exerted through an increased peripheral tissue glucose uptake and suppressed hepatic gluconeogenesis (Lam et al. 2012). Borapetoside C **(14)** (0.1 mg/kg) is also capable of increasing glycogen synthesis in skeletal muscles when given in combination with insulin in normal, type-1 and type-2 diabetic mice. It increased the serine phosphorylation of Akt, phosphorylation of the insulin receptor, and GLUT2 levels by 3.0, 1.4 and 1.3-fold when administered with insulin (Ruan et al. 2012). This demonstrated the versatility of this compound in terms of antidiabetic activity. Another compound with established insulin secretagogue activity is borapetol B **(16)**, which was assessed on normoglycemic Wistar and spontaneously type-2 diabetic Goto-Kakizaki (GK) rats at a dose of 0.1 mg/kg. In the Oral Glucose Tolerance Test (OGTT), a significant decrease in glucose levels was observed in both animal models. This compound also enhanced insulin secretion in isolated pancreatic islets (Lokman et al. 2013). In a later study, borapetoside C **(14)** (IC_50_ value of 0.527 ± 0.008 mg/mL) and 4-hydroxybenzaldehyde **(130)** (IC_50_ value of 0.557 ± 0.004 mg/mL) were found to be the most potent α-glucosidase inhibitors. The alkaloids liriodenine **(49)**, lysicamine **(50)** and *N*-formylanonaine **(39)** also strongly inhibited this enzyme, with IC_50_ values ranging from 0.5 to 0.8 mg/mL. Borapetoside C **(14)** (IC_50_ value of 0.775 ± 0.005 mg/mL) displayed the most prominent activity against α-amylase alongside notable activity observed for *N*-*trans*-feruloyltyramine **(62)**, dihydrodiscretamine **(53)** and magnoflorine **(51)** (IC_50_ value of 0.8 to 0.9 mg/mL). It was suggested that the ring hybridization of these alkaloids allowed them to interact with the aforementioned enzymes, but that the presence of different functional groups weakened their activity (Hamid et al. 2015). Another clerodane furanoditerpenoid, borapetoside E **(4)** (40 and 80 mg/kg), caused stark improvements in hyperglycemia, insulin resistance, hyperlipidemia, hepatic steatosis and oxygen consumption in high fat diet-fed mice compared to the standard metformin (400 mg/kg). This compound also reduced the expression of sterol regulatory element binding proteins (SREBPs), which are important transcription factors in lipid synthesis and have emerged as novel targets for the treatment of type-2 diabetes (Xu et al. 2017). Tinosporol A **(8)** induced dose-dependent hypoglycemic activity in type-1 diabetic ICR (Institute of Cancer Research) mice and type-2 diabetic db/db mice, although it was found that the type-1 model was more sensitive to this compound than the type-2 one (Gao et al. 2016). In a study investigating the α-glucosidase inhibitory activity of acylated glucosylflavones (tested at a concentration of 10 μg/mL), isovitexin-2"-(*E*)-*p*-coumarate **(89)** displayed maximum inhibition (IC_50_ value of 4.3 ± 1.4 µM) compared to the standard acarbose (IC_50_ value of 0.033 ± 0.006 µM) (Chang et al. 2015).

Some clinical studies have been conducted to evaluate the effect of *T. crispa* on healthy volunteers, on patients with diabetes and patients with high risks of developing diabetes. For example, a clinical study conducted in Thailand, showed that pre-prandial administration of *T. crispa* (250 mg capsule twice daily for two months) in patients with metabolic syndrome resulted in a steady decrease in fasting blood sugar and triglyceride levels (Sriyapai et al. 2009). Another study reported a remarkable reduction in plasma glucose levels following oral administration of *T. crispa* powder (6 g) to healthy subjects (Rattanajarasroj et al. 2004). In both studies, however, *T crispa* caused a noticeable increase in ALT and AST serum levels, implying possible hepatotoxicity (Sriyapai et al. 2009; Rattanajarasroj et al. 2004). Other clinical studies also indicated the increased risk of hepatotoxicity associated with *T. crispa* and/or concluded that there was no evidence to support to use of this plant for the treatment of diabetes (Sangsuwan et al. 2004; Klangjareonchai and Roongpisuthipong 2012). In depth details and discussions on the clinical studies involving *T. crispa* can be found under the ‘Clinical Trials’ section.

**Fig. 9:**
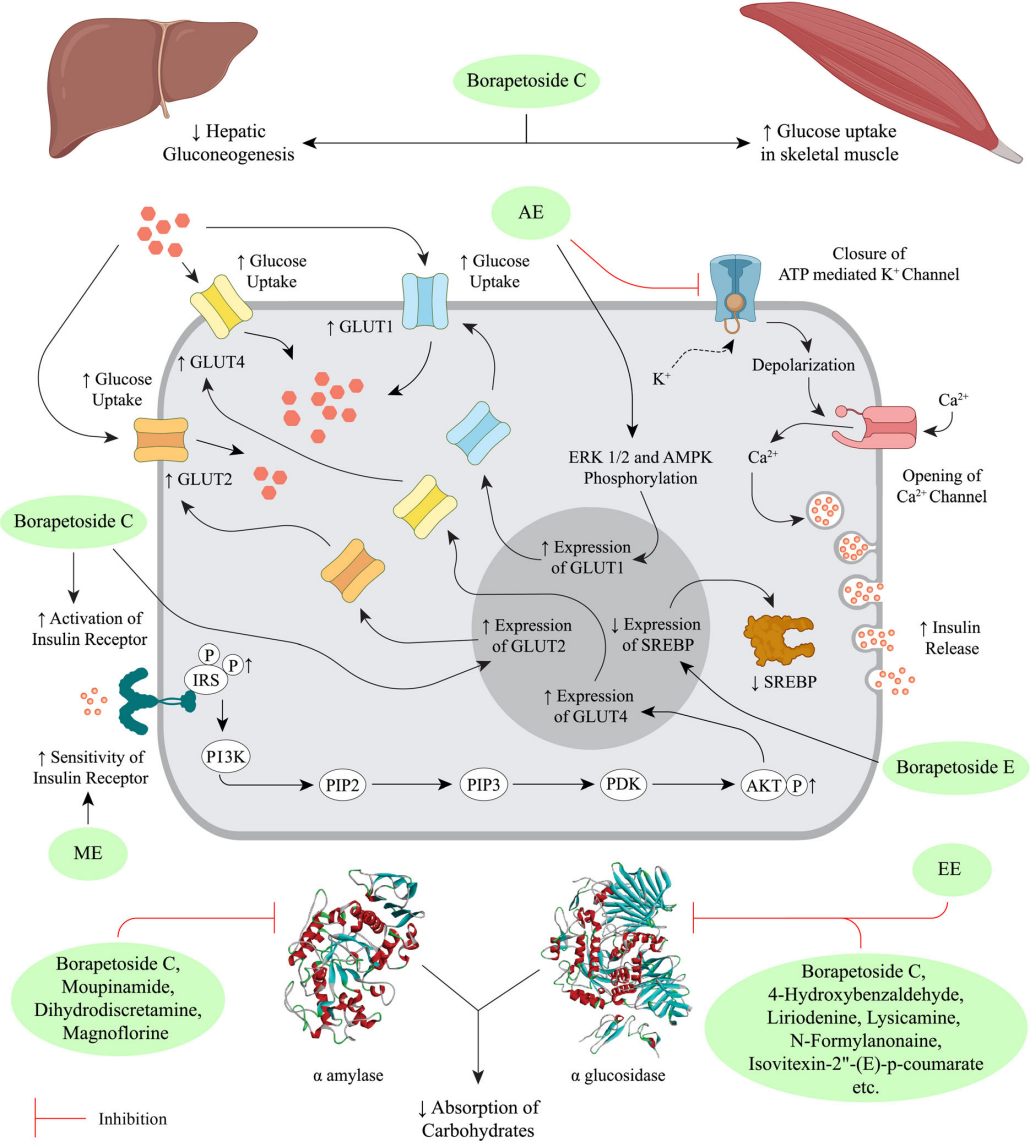
Schematic diagram of the antidiabetic mode of action of *Tinospora crispa*. *AE*: Aqueous Extract, *ME* Methanol Extract, *EE* Ethanol Extract, *ATP* Adenosine triphosphate, *GLUT* Glucose transporter, *ERK* Extracellular signal-regulated kinase, *AMPK* AMP-activated protein kinase, *IRS* Insulin receptor substrate, *P* Phosphate, *PI3K* Phosphoinositide-3-kinase, *PIP2* Phosphatidylinositol-4,5-bisphosphate, *PIP3* Phosphatidylinositol-3,4,5-trisphosphate, *PDK* Phosphoinositide-dependent kinase, *AKT* Protein kinase B, *SREBP* Sterol regulatory element-binding protein

In summary, the ethnomedicinal use of *T. crispa* in the treatment of diabetes has been underpinned by many scientific studies. The antihyperglycemic activity of this plant occurs mainly as a result of enhanced insulin secretion and inhibition of α- glucosidase and α-amylase. The pathways involved in the antidiabetic mode of action of *T. crispa* extracts and its phytoconstituents are similar (Fig. 9). Selected clerodane-type furanoditerpenoids present in *T. crispa* have been reported to possess insulin secretagogue properties. Further structure activity relationships (SAR) studies on this class of phytochemicals should be undertaken to determine the pharmacophore(s) responsible for the modulation of intracellular calcium ion levels. Other phytochemicals such as flavonoids, for example, have strong inhibitory activity against α-glucosidase and α-amylase and several SAR studies have been investigated these effects (Tadera et al. 2006; Proença et al. 2017, 2019; Zhu et al. 2020). Further research work on the antidiabetic potential of the various flavonoids present in *T. crispa* should be conducted.

### Cardiac activity and cardiovascular effects

Multiple extracts and fractions, at doses of 0.25–1 mg/mL, were evaluated for their cardioactive potential in isolated atria and aorta of male Sprague Dawley rats. Extraction was performed with petroleum ether, chloroform, methanol and water; and four fractions derived from the chloroform extract obtained following flash chromatography using chloroform/*n*-hexane and chloroform/methanol combinations. The fractions derived from the chloroform extract were found to be the most active, inhibiting the isoprenaline-induced positive chronotropic response in the left atrium by 80% at a dose of 1 mg/mL. From the dose–response curve obtained, it was concluded that all the extracts and fractions mentioned above functioned as non-competitive β-adrenoceptor antagonists. In the right atrium however, the extracts at high doses effectuated a complete inhibition of the isoprenaline-induced positive chronotropic response by suppressing the sinoatrial node. This could be rectified by high doses of isoprenaline. In the aorta, the fractions derived from the chloroform extract showed 85–99% inhibition of the noradrenaline-induced positive inotropic response, and the inhibition was commensurate with the increasing polarity of the fractions. The dose–response curve obtained suggested that these fractions acted as non-competitive α adrenoceptor antagonists (Bakhari and Isa 2010). The *n*-butanol fraction of the aqueous extract of *T. crispa* (1–100 mg/kg) was also tested in normal and reserpine-induced female Wistar rats. Whilst this fraction produced significant hypotensive and positive chronotropic activity in normal rats, dual effects were obtained following reserpine induction with a transient decrease followed by an increase in hypotensive activity. Similar dual effects were obtained for the positive chronotropic action. The mechanism of action was unravelled using post-treatment with propranolol (0.6 mg/kg), phentolamine (2 mg/kg), atenolol (2 mg/kg), the β2 antagonist ICI-118,551 (0.01 mg/kg), atropine (0.6 mg/kg) and hexamethonium chloride (10 mg/kg), either individually or in various combinations. This revealed that the action of the active constituents was mediated via β2-adrenergic receptors producing hypotension, as well as β1- and β2-adrenergic receptors effectuating a positive chronotropic response. Additionally, some constituents caused hypertension and increased heart rate via modulation of α-adrenergic receptors. The authors further concluded that compounds acting via non-adrenergic and non-cholinergic pathways were also present to cause a reduction in mean arterial pressure and heart rate (Praman et al. 2011).

Subsequent bioassay-guided fractionation resulted in the isolation of five cardio-active compounds from the *n*-butanol fraction, namely adenosine **(140)**, uridine **(142)**, salsolinol **(65)**, higenamine **(59)** and tyramine **(67)**. These compounds were assessed for their mechanism of action using the same model and chemicals including DMPX (an A2a adenosine receptor antagonist), suramin, phentolamine, ICI-118,551, atropine, prazosin and atenolol for post-treatment. Adenosine **(140)** (0.003–0.3 mg/kg) displayed hypotensive and negative chronotropic activity which was suppressed by DMPX. Uridine **(142)** (0.1–100 mg/kg) had a hypertensive and negative chronotropic effect in normal rats, which was inhibited by suramin. At high doses, it produced initial hypertension followed by hypotension. Salsolinol **(65)** (0.1–10 mg/kg) produced a hypotensive response with a decreased heart rate, which was suppressed significantly only by phentolamine. In reserpinized rats, however, hypertensive activity was observed for this compound, impeded by phentolamine, but not atenolol. Higenamine **(59)** (0.001–0.3 mg/kg) triggered hypotension in normal rats, which was obstructed by ICI-118,551 or atenolol. Similar results were observed in reserpinized rats, with prazosin increasing the hypotensive effect. Positive chronotropic effects were obtained in both animal models. Hypertension and increased heart rate were obtained in normal rats, but not in reserpinized ones, following treatment with tyramine **(67)** (0.003–1 mg/kg). The hypertensive effect dropped significantly by applying phentolamine, while the positive chronotropic effect was significantly boosted with atenolol. Salsolinol **(65)**, higenamine **(59)** and tyramine **(67)** were reported to exert their effects through the adrenergic pathway, while adenosine **(140)** and uridine **(142)** exerted their action via the purinergic pathway. All constituents acted in a dose-dependent manner (Praman et al. 2012). The compounds were further assessed for their inotropic action on isolated left atria using the same animal model. Adenosine **(140)** (10^−8^—3 × 10^−4^ M) and uridine **(142)** (10^−8^—10^−2^ M) acting via the purinergic pathway produced a negative and slightly positive inotropic effect, respectively. On the other hand, higenamine **(59)** (10^−8^–10^−5^ M), salsolinol **(65)** (10^−7^—10^−4^ M) and tyramine **(67)** (10^−8^—3 × 10^−5^ M) increased the force of contractility in the left atria via the adrenergic pathway. Additionally, salsolinol **(65)** at higher concentrations (3 × 10^−4^—3 × 10^−3^ M) induced a greater release of acetylcholine, leading to the opposite outcome (Praman et al. 2013).

**Fig. 10:**
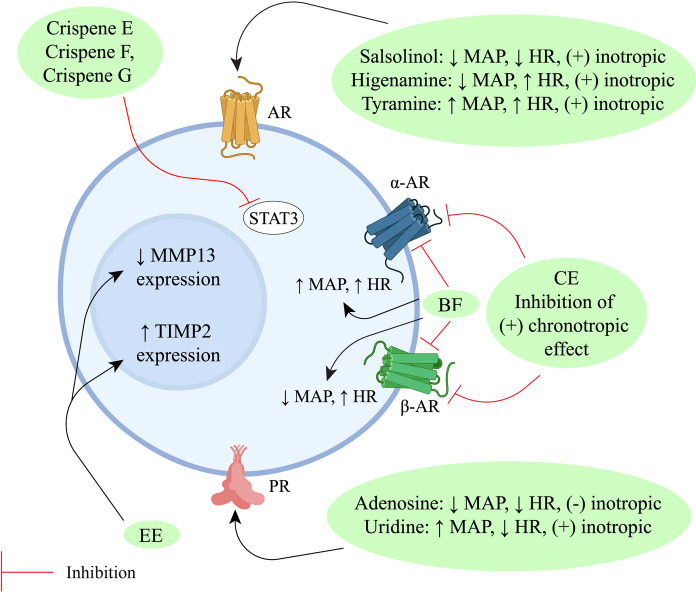
Schematic diagram of the cardioprotective and anticancer mode of action of *Tinospora crispa.* *CE* Chloroform Extract, *BF* *n*-Butanol Fraction, *EE* Ethanol Extract, *AR* Adrenergic Receptor, *PR* Purinergic Receptor, *MAP* Mean Arterial Pressure, *HR* Heart Rate, *STAT3* Signal Transducer and Activator of Transcription 3, *MMP13* Matrix Metalloproteinase 13, *TIMP2* Tissue Inhibitor of Metalloproteinases 2

Other compounds from *T. crispa* have been investigated for their cardio-active potential. This includes cycloeucalenol **(118)** (5.6 × 10^–5^ M) and cycloeucalenone **(119)** (5.6 × 10^–5^ M). Both molecules had slightly positive inotropic activity in the isolated right atria of male Wistar rats. Conversely, these compounds initially demonstrated minimal negative inotropic activity, followed by significant negative inotropic activity in the left atria, thereby exhibiting mild cardiotonic activity compared to noradrenaline (1 × 10^–8^ M) (Kongkathip et al. 2002). A synthetic racemic mixture of *N*-formylnornuciferine **(43)** produced a negative inotropic and chronotropic response in isolated rat heart (Imphanban et al. 2009). The identified mechanisms through which the *T. crispa* modulates cardio-activity are presented in Fig. 10. However, it should be noted that the cardiac potential of this plant cannot be attributed to a particular class of compounds with much confidence, other than the purinergic action of its nucleosides. Moreover, when administered to diabetic rats, *T. crispa* powder produced a significant increase in hemoglobin concentration and red blood cells (RBC) alongside a notable decrease in White Blood Cells (WBC) compared to control (Suchantabud et al. 2008).

### Anticancer activity

The cytotoxic potential of various extracts and fractions of *T. crispa* has been reported by multiple investigators using the brine shrimp lethality assay method. A petroleum ether fraction of the methanol extract was reported to have strong cytotoxic activity with IC_50_ of 173 ppm (Mackeen et al. 2000). Another study revealed that the methanol extract of the stem along with its chloroform and petroleum ether fractions at doses of 0.781–400 μg/mL showed comparable cytotoxicity (LC_50_ of 12.0, 11.5, and 12.6 μg/mL, respectively). Vincristine sulfate was used as a standard with an LC_50_ of 0.323 μg/mL (Haque et al. 2011). Stronger cytotoxicity (LC_50_ values of 6.43, 4.58, and 0.80 μg/mL, respectively) was later reported in another study on the same stem extract and fractions tested within the same concentration range. This study also evaluated the aqueous extract which showed a LC_50_ of 7.46 μg/mL (Islam et al. 2013). The ethanol extract of the leaves had a LC_50_ of 62.75 μg/mL, which is notably weaker compared to the previously mentioned extracts (Tarukbua et al. 2018). The methanol extract of the stems was found to suppress the proliferation of HL-60, HEP-G2 and Hep3B cancer cells in a dose- and time-dependent manner (Ahmad et al. 2016a). The aqueous extract showed moderate antiproliferative activity against MCF-7, Caov-3, HeLa and HEP-G2 cells (IC_50_ of 107, 100, 165 and 165 μg/mL, respectively) (Zulkhairi Jr et al. 2008). The aqueous, methanol and chloroform extracts of *T. crispa* stem revealed antiproliferative and cytotoxic activity against MCF-7, MDA-MB-231, 3T3 and HeLa cells. The extracts produced dose-dependent cytotoxicity, with the methanol extract being the most potent (Ibahim et al. 2011). The ethanol extract (12.5, 25, 50, and 100 μg/mL) showed inhibition of head and neck squamous cell carcinoma (HNSCC) metastasis on HN22 and HSC3 cells. In a 3-(4,5-dimethylthiazol-2-yl)-2,5-diphenyltetrazolium bromide (MTT) assay, this extract, at the maximum concentration used, significantly decreased cell viability in the two cell lines to 50% and 60%, respectively compared to the negative control dimethyl sulfoxide (DMSO). Administration of this extract at concentrations of 12.5, 25, and 50 μg/mL also downregulated MMP-13 gene expression in both cell lines. A stronger reduction in secreted MMP-13 levels was observed in HN22 compared to that of HSC3 cells. In the latter cell line, the ethanol extract at 25 and 50 μg/mL increased the expression of the tissue inhibitors of metalloproteinase-2 (TIMP-2). Moreover, pre-treatment with this extract (50 μg/mL) in a scratch wound healing assay using HN-22 cells caused cell migratory activity to drop to 65% compared to the control DMSO (Phienwej et al. 2015).

The chloroform extract of the stems was evaluated for its anti-angiogenic activity in the Chick embryo Chorioallantoic Membrane (CAM) induced by basic Fibroblast Growth Factor (bFGF) assay. Dose-dependent anti-angiogenic activity of 31.87 ± 9.01, 43.12 ± 8.01, 53.44 ± 2.70 and 62.81 ± 4.74% was obtained for concentrations of 15, 60, 240, and 960 μg/mL, respectively (Triastuti 2010). In contrast, no cytotoxic activity was reported for the methanol and aqueous extracts of the stems in a water-soluble tetrazolium (WST) or MTT assay employing HL-60, HEP-G2 and MCF-7 cancer cells (IC_50_ > 500 μg/mL) (Tungpradit et al. 2010). This apparent difference of activity on different cell lines may depend upon the nature of phytoconstituents present in the extracts. This, in turn, may be linked to differences in geographical areas of plant collection as has been reported previously when samples collected from different regions of the East Jawa province in Indonesia showed significant difference in cytotoxicity. The ethanol extract yielded LC_50_ values ranging from 30.64 ± 2.18 (strong activity) to 254.15 ± 30.77 μg /mL (weak activity) in an MTT assay carried out on MCF-7 breast cancer cells (Mutiah et al. 2019).

Tinocrisposide **(14)** (3.125–100 μg/mL) isolated from the dichloromethane fraction of the methanol stem extract was tested using an MTT assay on H1299 and MCF-7 cells. IC_50_ values of 70.9 and > 100 μg/mL were obtained in these cell lines, respectively. It was suggested that this compound, whilst not a viable cytotoxic agent, could still prove useful as a chemopreventive agent (Adnan et al. 2016). The *cis*-clerodane furanoditerpenoid crispene E **(10)** isolated from the *n*-hexane fraction of the methanol stem extract exerted notable inhibition of Signal Transducer and Activator of Transcription Protein 3 (STAT-3) both in a fluorescent polarization (FP)-based primary protein–protein binding assay and a MTT assay. In the FP assay, this compound exhibited an IC_50_ of 10.3 μM and 210% inhibition relative to the STAT-3 SH2 domain interacting molecule STA-21. The mentioned domain is pivotal for dimerization, which is in turn implicated in the development of different cancers. The IC_50_ values for the HeLa (cervical), MIA PaCa2 (pancreatic), NCI H1975 (non-small cell lung), MDA-MB-231 (breast) cancer cell lines in the MTT assay were 10.5, 8.3,11.8 and 5.4 μM, respectively (Mantaj et al. 2015). A subsequent study isolated two related compounds, crispene F **(2)** and crispene G **(11)**, which yielded IC_50_ values of 42 and 17 μM, respectively, in the FP assay and 119% to 130% inhibition compared to STA-21, respectively. Both compounds had IC_50_ values of 10 and 7.8 μM on MDA-MB-231 cells using the MTT assay. Weak activity on A4 (STAT-3 independent) colon cancer cells indicated that the compounds possibly induced STAT-3-specific inhibition. Comparatively, crispene E **(10)** was identified as the most potent among the three derivatives (Noman et al. 2018).

The in vitro anticancer activity of *T. crispa* has been demonstrated against several cancer cell lines. Its effects on gene expression and the underlying mechanisms are illustrated in Fig. 10. There have been no studies reported on the anticancer activity of the plant in vivo, which warrants further investigations. Interestingly, pure compounds such as clerodane-type furanoditerpenoids have displayed promising activity, particularly on STAT-3 inhibition. Quantitative SAR (QSAR) studies are now required into the 38 compounds of this class that have been isolated from the plant. This may help to focus on specific chemical moieties that can interact with the binding sites of interest in the STAT-3 protein.

### Antiparasitic activity

Although *T. crispa* has been reported as a traditional medicine against parasites, particularly *Plasmodium* (Vigneron et al. 2005; Malik 2015), investigations carried out to date have provided conflicting accounts on its antimalarial activity. The methanol stem extract (dose of 0.1–2.5 mg/mL) was evaluated for in vitro antiplasmodial activity against *Plasmodium falciparum* (FCR-3 strain). The highest dose of this extract showed 100% inhibition after 24 h of incubation. In vivo activity was further studied in adult female mice infected with *Plasmodium berghei* (chloroquine sensitive ANKA strain). At a dose of 5 mg/kg, the extract led to 0–32.7% parasitemia from days 1 to 5 post-infection, which was lower than the negative control. However, antiplasmodial activity was not considered to be significant (Rahman et al. 1999). Similarly, inconsequential results were obtained in another study testing the same extract against the same strain (Niljan et al. 2014). *Tinospora crispa* aqueous extract (1 mg/mL) yielded approximately 40% inhibition of *P. falciparum* and 80% inhibition of *Babesia gibsoni* in infected erythrocytes. In case of *P. falciparum*, the extract was considered to be inactive (Murnigsih et al. 2005). Similar inactivity against *P. falciparum* was also observed for the ethanol, ethyl acetate and *n*-hexane fractions of *T. crispa* stems (Ramadani et al. 2018). The methanol extract (0.5–3.0 mg/mL) showed IC_50_ values between 0.27–0.29 mg/mL against *P. falciparum* 3D7 strain. Artemisin was used as a standard and showed an IC_50_ of 10^–8^ mg/mL. The 2 mg/mL dose was found to significantly lower the parasitic load, with the percentage parasitemia and parasite DNA concentration reduced by 47.12% and 56.83%, respectively. At doses above 2.0 mg/mL, these effects did not correlate with the dose administered. It was postulated that antioxidant activity was responsible for the observed effects (Ihwan et al. 2014). In a different study using the same model, the ethanol extract was found to be more potent (IC_50_ of 0.344 ± 0.210 µg/mL). In the in vivo study using male Swiss mice infected with *P. berghei* NK65, the extract (doses of 50–400 mg/kg) had an ED_50_ of 271.89 ± 4.32, and consequently the plant was deemed to possess moderate activity (Abdillah et al. 2015). In another in vitro assay, the methanol extract displayed an EC_50_ value of 7.5 µg/mL, indicating strong antimalarial activity (Tran et al. 2003). The ethanol extract when administered at doses of 20, 40 and 80 mg/kg to ICR mice infected with *P. yoelii* 17XL demonstrated dose-dependent activity, with 53.68% parasitemia on day 18 at the highest dose (Rungruang and Boonmars 2009). In another assay using ICR mice infected with *P. berghei* (ANKA strain), 13-hydroperoxyoctadeca-9,11-dienoic acid **(159)** was identified as a probable antimalarial compound (Lee et al. 2020). The aqueous extract of the plant also exerted hepatoprotection in ICR mice infected with *P. berghei*. The liver damage, indicated by increased serum alanine aminotransferase (ALT) and aspartate aminotransferase (AST) levels, was inhibited by this extract at a dose of 500 mg/kg (Somsak et al. 2015). In the same model, the aqueous extract at doses of 500, 1000 and 2000 mg/kg displayed renoprotective and antihemolytic effects. At higher doses, the blood urea nitrogen (BUN) and creatinine levels decreased significantly compared to the negative control. For the highest dose, the hematocrit percentage increased significantly compared to the untreated group (Nutham et al. 2015).

Three combinations of artesunate (32 mg/kg) were prepared using three doses of the aqueous extract (2.5, 3 and 3.5 mg/kg) and administrated to C57BL/6 J mice infected with *P. berghei*. This caused a substantial inhibition of Nuclear Factor Kappa B (NFκB) and Intracellular Adhesion Molecule-1 (ICAM1) compared to the artesunate or extract only groups (Izzati et al. 2016). The aqueous extract of *T. cripsa* stems was also assessed against *Brugia malayi,* amongst other parasites, to evaluate its antifilarial potential. Following an incubation period of 24 h, the extract produced relative mobility values of 25, 7 and 0 at doses of 1, 5 and 10 mg/mL, respectively (Zaridah et al. 2001). Another study reported that an ointment prepared from an oil extract of the stem displayed significant activity against *Pediculus humanus capitis* compared to a shampoo used as a positive control and containing 1% permethrin (Torre et al. 2017). The ethanol extract of the stem (1.56–200 μg/mL) also proved to be active against *Toxoplasma gondii* (RH strain) compared to standards of veratrine and clindamycin used at the same concentrations. This extract did not display any cytotoxicity in an MTT assay against Vero cells (IC_50_ value 179 μg/mL) compared to clindamycin (IC_50_ of 116.5 μg/mL) and veratrine (IC_50_ of 60.4 μg/mL). The antitoxoplasmic activity of the extract was established with an IC_50_ of 6.31 μg/mL compared to that of clindamycin (8.33 μg/mL) and veratrine (14.25 μg/mL). The good selectivity index calculated for this extract (28.4) suggests it may represent a promising source of new antitoxoplasmic agents (Sharif et al. 2019).

Overall, *T. crispa* has demonstrated in vitro and in vivo activity against various parasites, but there have been contradictory reports regarding the potency of its extracts against *Plasmodium* species. Further pharmacological investigation and bio-assay guided isolation of active compounds are required in the future.

### Antimicrobial activity

An in vitro disk diffusion assay was carried out to evaluate the antimicrobial activity of the aqueous, ethanol and chloroform extracts of *T. crispa* (25, 50, 75, and 100%) against various Gram- positive (*Staphylococcus aureus, Streptococcus pneumoniae, Corynebacterium diphtheriae, Bacillus cereus, Listeria monocytogenes*) and Gram-negative (*Escherichia coli, Salmonella typhi, Shigella flexneri, Klebsiella pneumoniae, Proteus vulgaris*) bacteria using flemequine as a standard. All extracts dose-dependently inhibited *S. pneumoniae, C. diphtheriae* and *S. flexneri* compared to the standard. At concentrations above 50%, the aqueous and chloroform extracts inhibited *S. aureus and E. coli*. All extracts were ineffective against *B. cereus* and *S. typhi* (Zakaria et al. 2006). Additional testing of the aqueous extract on *S. aureus* and *E. coli* using an agar diffusion assay, led to a modest inhibitory effect with Minimum Inhibitory Concentration (MIC) and Minimum Bactericidal Concentration (MBC) of 227.27 mg/mL each (Zakaria et al. 2011). Another study showed that the aqueous, ethanol, methanol and chloroform extracts of the plant were active against *S. pneumoniae, E. coli* and *Candida albicans* compared to the standards tetracycline and fluconazole (Asif Iqbal et al. 2012). The ethanol extract at a dose of 1 mg/disk was also active against Methicillin Resistant *S. aureus* (MRSA) compared to the standard vancomycin in a disk diffusion assay (Al-alusi et al. 2010). Furthermore, the ethanol extract, when administered as ointment (9% v/v) with zeolite, showed bactericidal activity against *S. aureus* and *Pseudomonas aeruginosa* compared to a preparation containing gentamicin (Susanti et al. 2016). Another disk diffusion assay study confirmed the efficacy of the ethanol extract against *E. coli* (zone of inhibition of 20–22 and 22–30 mm at concentrations of 8% and 32%, respectively) compared to the standard amoxicillin (19 mm) (Muslimin et al. 2018). The aforementioned extract also showed strong antifungal activity against *Trichophyton rubrum* at concentrations ≥ 40% (Erza et al. 2020). The *n*-hexane extract of the stem significantly inhibited the growth of *S. aureus*, *Shigella boydii, S. dysenteriae, Vibrio mimicus, C. albicans* and *Aspergillus niger* (Rahman et al. 2020). Two oxaporphine alkaloids isolated from the plant, namely lysicamine **(50)** and liriodenine **(49),** displayed activity on *S. aureus* and *Enterococcus faecalis* in a disk diffusion assay (Hamid et al. 2021). The plant ethanol extract, when employed as a 30% ointment, also revealed activity against *Propionibacterium acnes* (zone of inhibition of 9.13 mm), indicating its potential as an anti-acne treatment (Yusriani et al. 2018). One study tested the chloroform and petroleum ether fractions of the methanol extract of *T. crispa* using a disk diffusion assay against five Gram-positive bacteria (*Bacillus subtilis, B. megaterium, B. cereus, S. aureus, Sarcina lutea*), seven Gram-negative bacteria (*E. coli, S. dysenteriae, S. typhi, S. paratyphi, S. boydii, V. mimicus, V. parahemolyticus*) and three fungi (*C. albicans, A. niger and Sacharomyces cerevisiae*). The activity of the extract and fractions (400 μg/disc) was compared to that of the standard doxycycline (30 μg/disc). Zones of inhibition, albeit negligible, were only observed for the chloroform fraction (Haque et al. 2011). The weak activity of the chloroform fraction was confirmed by another study testing the same fractions against the aforementioned microorganisms and *P. aeruginosa*, and using kanamycin (30 μg/disc) as a standard. This study reported no activity for the petroleum ether fraction (Islam et al. 2014). The antibacterial activity of the protein extract of *T. crispa* was evaluated against *B. cereus, S. aureus, K. pneumoniae and Salmonella typhimurium*. Only *B. cereus* was found to be sensitive to the extract (zone of inhibition of 9.7 ± 0.5 mm) (Zin et al. 2016).

The antiviral activity of *T. crispa* was evaluated for the ethanol and aqueous extracts (3–100 µg/mL) against HIV-1 integrase. Weak activity was obtained (IC_50_ > 100 µg/mL) (Bunluepuech and Tewtrakul 2009). Another study reported the use of a molecular docking approach to investigate the interactions of a variety of *T. crispa* constituents (putatively detected by GC–MS) with the SARS-CoV2 main protease. Imidazolidin-4-one and 2-imino-1-(4-methoxy-6-dimethylamino-1,3,5-triazin-2-yl) **(64)** were found to bind with the active site of this enzyme in a similar manner to the standard nelfinavir (Rakib et al. 2020c).

Overall, *T. crispa* extracts have demonstrated in vitro activity against selected microorganisms, which should be further investigated particularly employing in vivo models of infection. Also noteworthy are bioassay-guided studies to identify the phytoconstituents responsible for such activity. Hamid et al. (2021) have reported that aporphine alkaloids had good activity against Gram-positive bacteria. A total of 13 alkaloids of this type have been isolated from *T. crispa* to date, warranting further testing and SAR studies. The molecular mechanisms underlying the antimicrobial activity of *T. crispa* extracts/constituents should also be elucidated. Considering the current global antimicrobial drug resistance issue, unravelling the specific microbial pathway(s) targeted and the chemical pharmacophores are particularly important as this may pave the way for future antibiotic design and development.

### Immunomodulatory activity

The ability of *T. crispa* to modulate the innate and adaptive immune response has been demonstrated in several studies. The plant contains both anti-inflammatory and pro-inflammatory constituents. In the carrageenan-induced rat paw oedema model, the methanol extract of the stem at a dose of 10 mg/kg produced a 38% suppression of the oedema. The *n*-butanol fraction of the same extract was more effective than the diethyl ether and the aqueous fractions. When administered subcutaneously a dose of 3 mg/kg, the *n*-butanol fraction showed activity comparable to 250 mg/kg sulpyrine and 10 mg/kg diphenhydramine (Higashino et al. 1992). The anti-inflammatory activity of the plant was also assessed using an antigen-induced rat basophilic leukemia (RBL)-2H3 cell line where release of β hexoaminidase was measured. The ethanol extract and aqueous extract of the stem (concentration range of 0–100 μg/mL) revealed dose-dependent inhibition up to 44% and 65%, respectively. However, their IC_50_ values were higher (> 100 μg/mL and 83 μg/mL, respectively) compared to the standard ketotifen fumerate (20.2 μg/mL), suggesting weak activity. Interestingly, the ethanol extract of *T. crispa* combined with the ethanol extract of *Piper nigrum* (1:1, v/v) produced an IC_50_ of 26.7 μg/mL (Kraithep et al. 2008). The methanol extract was evaluated for its ability to inhibit reactive oxygen species (ROS) in whole blood, polymorphonuclear (PMN) leukocytes and macrophages during phagocytosis using a luminol/lucigenin-based chemiluminescence assay. The extract produced significant suppression of ROS in the metabolic phase of phagocytosis (IC_50_ of 0.6 ± 4.2 μg/mL compared to 3.0 ± 1.3 for the standard acetylsalicylic acid). It performed poorly in the other assays that were used in the study, including the PMN chemotaxis assay, compared to the standard ibuprofen (Jantan et al. 2011). Another study involving both the methanol and aqueous extracts of *T. crispa* stem was carried out on hydrogen peroxide-induced human umbilical vein endothelial (HUVEC) cells using a Tumor Necrosis Factor-α (TNF-α)-induced model of inflammation. The extracts inhibited Intracellular Adhesion Molecule- 1 (ICAM-1), Vascular Cell Adhesion Molecule-1 (VCAM-1) in a dose-dependent manner at concentrations ranging from 100–600 μg/mL. A significant and dose-dependent increase in Nitric Oxide (NO) production was observed in the presence of both extracts (Kamarazaman et al. 2012). In the carrageenan-induced paw oedema model, the aqueous extract of *T. crispa* (50, 100 and 150 mg/kg) showed inhibition comparable to ibuprofen (0.5%). In an in vitro membrane stabilization assay using hypotonic solution-induced lysis of human RBCs, the extract at a concentration of 2.5% was not active. At concentrations of 5 and 7.5%, however, it showed membrane stabilization comparable to ibuprofen (0.5%). The extract also dose-dependently inhibited the denaturation of protein in an albumin solution (Hipol et al. 2012). The ethanol extract (50, 100 and 200 mg/kg) was also tested on male Balb/C mice primed with sheep RBCs, using levimasole as a positive control. The results indicated that this extract increased peritoneal macrophage engulfment of *E. coli*, NO production, and lysozyme and myeloperoxidase serum levels. The extract at a dose of 200 mg/kg was equivalent to 2.5 mg/kg of levimasole. Upregulation of Immunoglobulin G (IgG) and Immunoglobulin M (IgM) also occurred, with the extract at the dose 100 mg/kg proving more potent than the standard. Dose-dependent delayed hypersensitivity was also observed in a footpad edema assay (Ahmad et al. 2016b).

A number of studies succeeded in elucidating the active constituents and their biological potential in immunomodulatory assays. Using a flow cytometry immunostaining assay on lipopolysaccharide (LPS)-induced RAW 264.7 cells, *T. crispa* ethanol extract and fractions were found to considerably boost the levels of the pro-inflammatory cytokines Interferon γ (IFN-γ), Interleukin 6 (IL-6) and IL-8. Cordioside **(13)**, quercetin **(82)**, eicosenoic acid (paullinic acid) **(160)** and boldine b were isolated from a fraction coded as Fraction 2 (Abood et al. 2014). In a chemotaxis assay carried out on RAW 264.7 cells with the chemoattractant formyl-methionylleucyl-phenylalanine, the ethanol extract (12.5–200 μg/mL) increased chemotaxis as compared to the standard. Compounds from the ethanol extract which displayed notable immunomodulatory activity were identified as *N*-formylanonaine **(39)**, *N*-formylnornuciferine **(43)**, lysicamine **(50)**, magnoflorine **(51)**, syringin **(134)** and 1-octacosanol **(167)**. When tested in the chemotaxis assay at concentrations ranging from 1.56–25 μg/mL, the first four compounds—particularly magnoflorine **(51)**—showed a potentiating effect, while the last two—particularly syringin **(134)—**inhibited chemotaxis compared to the standards ibuprofen and levimasole. ROS production, phagocytosis, NO, prostaglandin E_2_ (PGE_2_), Monocyte chemoattractant protein-1 (MCP-1), IL-6, IL-1β and TNF-α levels were also boosted by the extract, magnoflorine **(51)**, *N*-formylanonaine **(39)**, *N*-formylnornuciferine **(43)** and lysicamine **(50)**. Magnoflorine **(51)** proved to be most potent in this regard. Opposite effects were found for syringin **(134)** and 1-octacosanol **(167)**. It was concluded that among the compounds tested, syringin **(134)** and 1-octacosanol **(167)** showed anti-inflammatory properties, while the rest activated the immune system (Ahmad et al. 2018). Magnoflorine **(51)** and syringin **(134)** were further confirmed to be important immunomodulatory constituents of the ethanol extract. In LPS-primed U937 human macrophages, both the ethanol extract and magnoflorine **(51)** enhanced Inhibitory κB Kinase (IKK) α/β and NFκB phosphorylation while simultaneously causing de-activation of IκBα. Subsequently, activation of NFκB occurred alongside release of IL-1β and TNF-α. In addition to this, the extract resulted in the upregulation of cyclooxygenase-2 (COX-2) and PGE_2_ along with phosphorylation of Akt, extracellular signal-regulated kinase (ERK) 1/2, p38 mitogen-activated protein kinase (MAPK), and c-Jun N-terminal kinase (JNK) 1/2 (Haque et al. 2020). Tinocrisposide **(14)** (100–1000 μg/mL) was another compound assayed for its hemolytic and anti-inflammatory potential. Its hemolytic value (< 10%) suggested it was non-hemolytic. Moreover, in an in vitro anti-inflammatory assay, this compound displayed membrane stabilizing activity comparable to the standard ibuprofen. Similar results were obtained for the aqueous extract of the plant (Adnan et al. 2019).

A recent in silico study postulated that tyramine **(67)** may act as a COX-2 inhibitor and exert anti-inflammatory activity (Widodo et al. 2021). While it is confounding that *T. crispa* phytoconstituents are able to both activate and suppress the immune system, it also opens up possibilities into designing new classes of immunomodulators. It is noticeable that the compounds of interest are not confined to a particular chemical class. This may also explain the marked diversity in the biochemical responses produced.

### Antioxidant activity

The antioxidant activity of various extracts and fractions of the plant has been studied extensively. In this regard, the methanol extract was found to be more potent compared to the aqueous and chloroform extracts. In a 2,2-diphenyl-1-picrylhydrazyl (DPPH) free radical scavenging assay, the methanol extract had an IC_50_ of 12 μg/mL which was comparable to the standard ascorbic acid. The resultant inhibition also approached 100%. Its total phenolic and flavonoid contents were found to be 255.33 ± 10.79 mg Gallic Acid Equivalent (GAE)/g sample and 9.53 ± 0.50 mg Quercetin Equivalent (QE)/g sample, respectively (Ibahim et al. 2011). Another study used a DPPH free radical scavenging assay on the ethanol extract, aqueous fraction and ethyl acetate fraction. The ethyl acetate fraction displayed the strongest activity (53.77% inhibition at 200 μg/mL) (Irianti et al. 2011). Several in vitro and in vivo studies were performed on the aqueous extract. The latter at a concentration of 10% produced DPPH inhibition, Thiobarbituric Acid (TBA) inhibition and displayed a Ferric Reducing Antioxidant Power (FRAP) value of 86.51 ± 0.07%, 39.2 ± 5.14% and 0.89 ± 0.07 mmol/L, respectively compared to the standards ascorbic acid (96.36 ± 0.90%, 73.2 ± 5.14% and 1.05 ± 0.00 mmol/L, respectively) and butylated hydroxytoluene (96.51 ± 0.95%, 75.8 ± 6.08% and 1.03 ± 0.03 mmol/L, respectively). An in vivo study was carried out on hypercholesterolemic rabbits using the aqueous extract at doses of 150, 300 and 450 mg/kg. The extract reduced Total Cholesterol (TC), Triglyceride (TG) and Low-density Lipoprotein (LDL) while boosting High-density Lipoprotein (HDL) and restored malondialdehyde (MDA) levels to normal. Aortic atherosclerotic lesions were dose-dependently lessened up to 100%. This suggests that the antioxidant potential of *T. crispa* is linked to its inhibition of atherosclerosis and plasma lipid peroxidation (Amom et al. 2011). The aqueous extract of *T. crispa* stem showed anti-atherosclerotic and anti-hypercholesterolemic activity in adult male New Zealand albino rabbits. The animals were first conditioned with a 0.5% high cholesterol diet, which caused an increase of C-Reactive Protein (CRP) levels. A dose-dependent reduction of CRP levels was observed following administration of the extract. At 200 mg/kg, the extract did not change the CRP levels. At 450 mg/kg, it returned the CRP levels to normal levels while at 600 mg/kg it reduced the CRP levels to levels lower than normal. The extract also dose-dependently reduced atherosclerotic plaque coverage and foam cell formation to a considerable degree (Shah et al. 2021). Further investigations were carried out on the radical-scavenging activity of the methanol extract and its petroleum ether, chloroform, carbon tetrachloride and aqueous fractions, using a DPPH assay. The carbon tetrachloride fraction showed the strongest activity with an IC_50_ value of 30 μg/mL compared to the standard ascorbic acid (15 μg/mL) and BHT (25 μg/mL) (Haque et al. 2011). In another study using a DPPH assay, the ethanol extract, its water fraction and selected subfractions, showed IC_50_ values of 49.92 μg/mL, 38.25 μg/mL, 36.12 μg/mL, and 16.18 μg/mL, respectively. It was postulated that acid hydrolysis of the subfractions improved their antioxidant potential (Warsinah et al. 2020). Several other studies measuring the total phenolic content, total flavonoid content, DPPH free radical scavenging activity and Ferric Reducing Antioxidant Power of *T. crispa* all confirned the antioxidant potential of the plant (Zulkefli et al. 2013; Abood et al. 2014; Nguyen et al. 2020; Mahalle and Gupta 2021). In a metal chelating assay, the petroleum ether, chloroform, methanol and water extracts of the stem were first mixed and dried together. The mixed extract (1 mg/mL) produced 81.97% inhibition of Ferrozine-Fe^2+^ complex formation compared to ethylenediaminetetraacetic acid (EDTA) at the same concentration (98.51% inhibition) (Zulkefli et al. 2013). In an MTT cell viability assay, pre-treatment with the aqueous extract (50–1000 μg/mL) and the methanol extract (600 μg/mL) of *T. crispa* boosted viability to 69% and up to 76%, respectively. When assessed for antioxidant activity in hydrogen peroxide-induced HUVEC cells, antioxidant enzymes including Catalase (CAT), Superoxide Dismutase (SOD) and Glutathione Peroxidase (GPx) were increased by the aqueous extract in a dose-dependent manner. The methanol extract on the other hand showed maximum CAT and SOD activity at 400 μg/mL and potentiated GPx activity dose-dependently. MDA levels were inhibited up to 58% and 60% for the aqueous and methanol extracts, respectively (Kamarazaman et al. 2012). A study using hyperlipidemic rabbits further confirmed the effect of the aqueous extract (administered at doses of 200, 450 and 600 mg/kg) on the cholesterol profile and the amelioration of atherosclerotic plaques compared to the standard simvastatin. Whilst SOD and GPx activity were also potentiated, the Total Antioxidant Status (TAS) did not improve substantially in the presence of *T. crispa* aqueous extract (Zamree et al. 2015). Three isolated constituents, *N-trans*-feruloyltyramine/moupinamide **(62)**, *N-cis*-feruloyltyramine **(63)** and secoisolariciresinol **(135)** displayed stronger antioxidant activity than the standard BHT in a DPPH free radical scavenging assay (Cavin et al. 1998). Other compounds such as protoberberine alkaloids isolated from the plant, namely columbamine **(54)**, dihydrodiscretamine **(53)** and 4,13-dihydroxy-2,8,9-trimethoxydibenzo[a,g]quinolizinium **(55)** showed IC_50_ > 500–800 μg/mL in a DPPH free radical scavenging assay (Hamid et al. 2021).

**Fig. 11:**
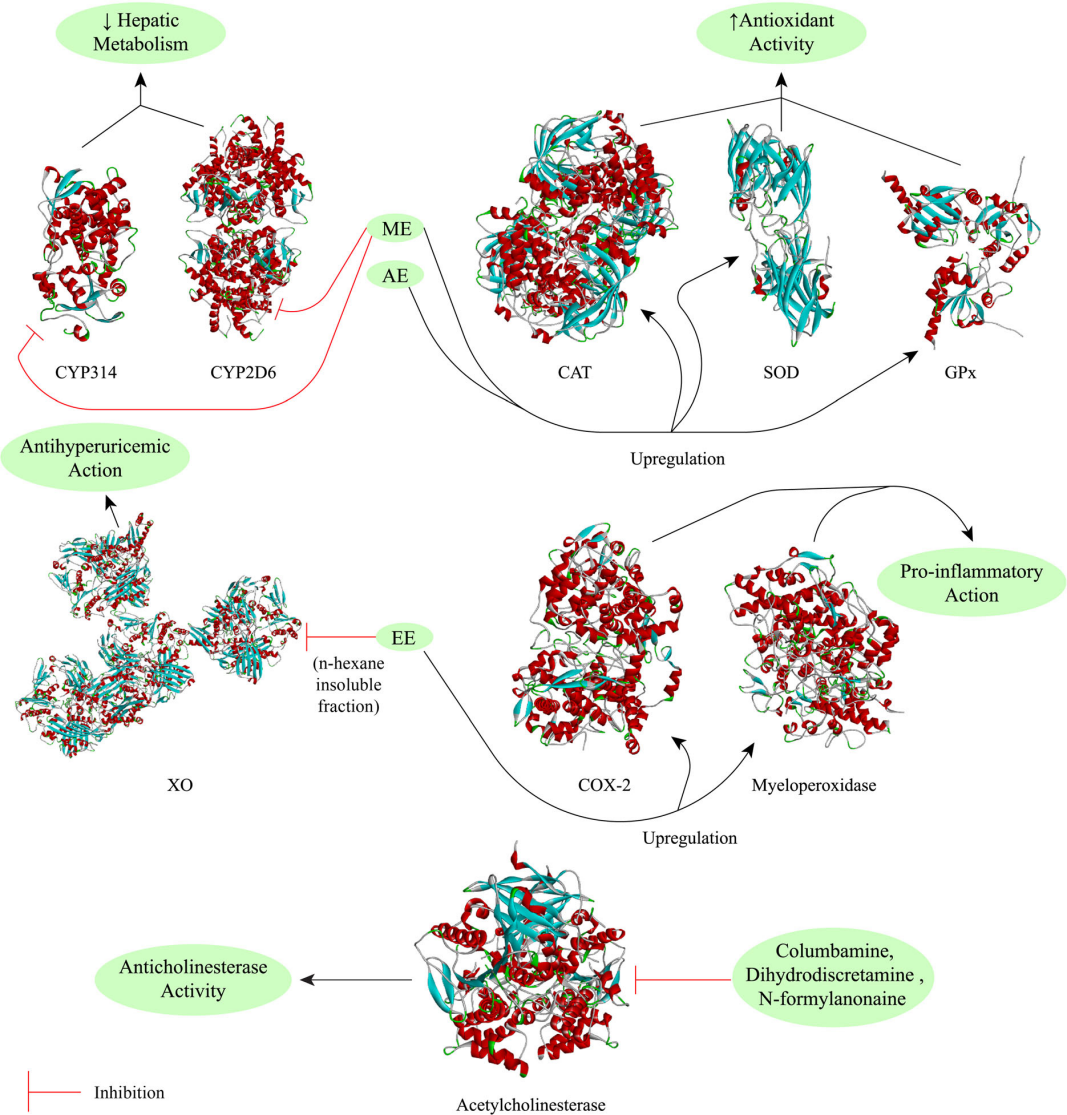
Schematic diagram of the modulation of miscellaneous enzymes by *Tinospora crispa*. *AE* Aqueous Extract, *EE* Ethanol Extract, *ME* Methanol Extract, *CAT* Catalase, *SOD* Superoxide dismutase, *GPx* Glutathione Peroxidase, *COX-2* Cyclooxygenase-2, *XO* Xanthine Oxidase

Whilst the antioxidant potential of *T. crispa* has been established in multiple in vitro studies, further in vivo studies are warranted, particularly focussing on how *T. crispa* extracts/constituents may interfere with antioxidant enzymes (Fig. 11). The numerous flavonoids present in the plant may contribute to the modulation of these enzymes, but this has yet to be assessed. Alkaloids, of the protoberberine class and others present in the plant should also be evaluated for their antioxidant potential so as to gain valuable insights into structure–activity relationships.

### Hepatoprotective activity

The hepatoprotective potential of *T. crispa* has been demonstrated in many studies (Lee et al. 2017; Rakib et al. 2020a). The methanol extract of *T. crispa* was found to increase the activity of phase-1 metabolic enzymes in male Sprague Dawley rat hepatocytes. The extract produced a substantial increase in aminopyrine *N*-demethylase activity at a dose of 0.001–1.0 mg/mL. At lower (but not higher) doses, this effect was mediated by the cAMP pathway (Tin et al. 2005). In an in vitro study, the same extract (0.5 mg/mL) produced 61.3% inhibition of the CYP3A4 enzyme compared to the standard troleandomycin (62.1%) in a time-dependent manner (Usia et al. 2006) (Fig. 11). The activity of this extract on CYP3A4 and CYP2D6 yielded IC_50_ values of 428 and 488 μg/mL, respectively (Subehan et al. 2006). The ethanol extract also acted against *tert*-butyl hydroperoxide-induced hepatotoxicity in HEP-G2 cells (EC_50_ of 144.3 μg/mL). The underlying mechanism was established to be via the induction of Nrf2-mediated expression of HO-1 (Lee et al. 2017). Another study demonstrated that carbon tetrachloride-induced Swiss albino mice pre-treated with the methanol extract (doses of 100–400 mg/kg body weight) resulted in noteworthy hepatoprotection. Levels of ALT, AST, Alkaline Phosphatase (AP), Malondialdehyde (MDA) and total bilirubin were reduced comparably to the standard silymarin (Rakib et al. 2020a). The enzyme modulatory and hepatoprotective activity of *T. crispa* warrants further investigations. In particular, bio-assay guided isolation studies should be performed to assess the activity of phytochemicals.

### Analgesic activity

Although used traditionally for pain management, the analgesic activity of the plant is not well studied. An extract of *T. crispa* stems was reported to demonstrate central analgesic activity in a tail flick response to radiant heat (Almeida et al. 2001). The ethanol extract (300 mg/kg) showed dose-dependent peripheral analgesia with 92% inhibition in the acetic acid-induced writhing test in mice, compared to the standard acetyl salicylic acid (81% inhibition at 100 mg/kg) (Sulaiman et al. 2008). In the same assay, the methanol extract, its petroleum ether and chloroform fractions (400 mg/kg) yielded 48.06, 51.94 and 43.41%, respectively, compared to 65.12% inhibition for the diclofenac sodium standard (100 mg/kg). The activity of the petroleum ether fraction was considered statistically significant (p < 0.05) compared to the standard (Islam et al. 2014). The methanol extract and the chloroform fraction (at doses 200 and 400 mg/kg) also displayed significant antinociceptive activity in the acetic acid-induced writhing and formalin-induced paw-licking tests, compared to the standard diclofenac (Rakib et al. 2020b). Having said that, the analgesic potential of the plant still requires further exploration. Future work should focus on investigations that aim to identify the phytoconstituents responsible for such activity. Studies on the molecular mode of action of the analgesic constituents must also be undertaken.

#### Antipyretic activity

The *n*-butanol fraction of *T. crispa* stems (3 mg/kg) suppressed LPS-induced fever in rats when administered intravenously. The activity was equivalent to that of 100 mg/kg sulpyrine and 1 mg/kg morphine hydrochloride administered intraperitoneally (Higashino et al. 1992). In DPT (Diphtheria-Pertussis-Tetanus) vaccine-induced male Wistar rats, a 40% ethanol extract of the plant produced significant antipyretic effect at 90- and 120-min post-treatment (Wulandari and Bestari 2016). Significant antipyretic activity was also observed for a methanol extract and its petroleum ether and *n*-hexane subfractions administered at a dose of 400 mg/kg to Swiss albino mice with Brewer’s Yeast-induced fever. The activity was found to be dose-dependent (Rakib et al. 2020a). These studies provide some evidence to support the ethnomedicinal use of *T. crispa* for the treatment of pyrexia. The specific molecular mode of action of such effects, however, remains to be elucidated.

#### Anticholinesterase activity

It is interesting to note that quaternary alkaloids are prevalent in *T. crispa*, indicating its probable acetylcholinesterase (AChE) inhibitory potential. One study assessed the potential of such alkaloids using a modified Ellman’s colorimetric method with physostigmine as the standard. Among the seven alkaloids studied, the least polar one—columbamine **(54)**—displayed significant inhibitory activity with an IC_50_ of 48.1 ± 1.3 μM compared to physostigmine (31.4 ± 0.5 μM). Dihydrodiscretamine **(53)** and *N*-formylanonaine **(39)** only showed moderate activity **(**Fig. 11). A preliminary SAR study was also performed on these alkaloids (Yusoff et al. 2014). QSAR studies employing the crystallized protein structure of AChE should be performed to gather information on the probable interactions of this target with bioactive ligands.

#### Central nervous system (CNS) activity

The activity of *T. crispa* on the CNS has not been studied extensively. A decoction of the plant was evaluated in a motor activity test, curiosity test, hanging test and rotary road test at various concentrations (6.5, 13 and 26%). It was found that the lowest concentration produced CNS-stimulant effects similar to the positive control caffeine (Merwanta et al. 2019). The methanol extract, its chloroform and *n*-hexane fractions at doses of 200 and 400 mg/kg were evaluated in the open field, hole board and elevated plus maze tests. A significant decrease in locomotion was observed in the open field test comparable to the standard diazepam (1 mg/kg). In the hole board test, the chloroform fraction at the highest dose yielded significant results, which indicated a reduced fearfulness. Additionally, the methanol extract (at the highest dose) and the chloroform extract (at the lowest dose) displayed anxiolytic activity in the elevated plus maze test comparable to the standard diazepam (1 mg/kg) (Rakib et al. 2020b). Additional investigations on the CNS activity of *T. crispa* are warranted, particularly focusing on the identification of the phytochemical(s) responsible for such activity.

#### Antihyperuricemic activity

The *n*-hexane insoluble fraction of the ethanol extract of *T. crispa* stem was evaluated in male BALB/C mice for its potential xanthine oxidase (XO) inhibitory activity. The extract reduced the levels of uric acid ranging from 49 to 78% at doses of 50–200 mg/kg. Peak activity was observed at the 100 mg/kg dose compared to the standard allopurinol (10 mg/kg) (Harwoko and Warsinah 2020) (Fig. 11). These results contradict a previous study carried out using the root of the plant, which showed an IC_50_ of 370.35 µg/mL compared to the standard allopurinol (0.022 µg/mL) (Vikneswaran and Chan 2005). This may suggest that the presence of phytoconstituents with prospective XO inhibitory activity is localized in certain parts of the plant. However, it is premature to drawing any conclusion on this aspect without supplementary evidence. Further identification of the phytoconstituents involved in the modulation of this enzyme are warranted.

#### Pesticidal activity

There is some evidence that *T. crispa* possesses pesticidal activity, although this has not been investigated exhaustively. Its chloroform, ethanol, petroleum ether and ethyl acetate extracts have been evaluated against the Small Mottled Millow Moth (*Spodoptera exigua*) which infests spinach. It was observed that the ethanol and petroleum ether extracts (five sprays over five days) reduced the moth population by 61.2% and 51.6%, respectively, compared to standard cyperin (91.5%). The other extracts did not produce significant inhibition (Isa et al. 2013). A similar study was carried out on *Phyliotera sinuata ateph* infesting mustard plants using the same extracts. Here, the ethanol and ethyl acetate extract (at a concentration of 1 g/L) reduced the insect population by 88.73% and 83.66%, respectively, compared to the standard cyperin (79.44%). Eight compounds namely, 1,2-benzenedicarboxylic acid **(144)**, 2-propenoic acid, dodecyl ester **(162)**, ethyl pentadecanoate **(163)**, oxalic acid, decyl 2-ethylhexyl ester **(164)**, 1-tetradecanol **(165)** 1-eicosanol **(166)**, and 1-octacosanol **(167)** were isolated from the ethanol extract but their bioactivity was not evaluated (Nor Aziyah et al. 2014). The ethanol extract (0.312, 0.625, 1.25, 2.5 and 5%) was also found to have larvicidal activity against the diamondback moth (*Plutella xylostella*) with an IC_50_ of 0.894% (Suvannarat et al. 2015). Larvicidal activity was also demonstrated against *Culex quinquefasciatus* mosquito larvae. The petroleum ether extract (80–160 ppm) of *T. crispa* mature fruits showed LC_50_ values ranging from 79.58 to 127.19 mg/L during the 1^st^–4th instars of growth (Pal et al. 2016). Another study revealed that the aqueous extract (3.125, 6.25, 12.5, and 25 mg/L) of the stem produced LC_50_ values of 16.95 and 30.12 mg/L, respectively (Jiraungkoorskul 2019). Additionally, the chloroform, *n*-hexane, methanol, and aqueous extracts of the stem displayed time- and concentration-dependent molluscicidal activity on *Pomacea canaliculata*. The *n*-hexane, followed by the aqueous extract, were the least cytotoxic of all extracts tested. The chloroform and methanol extracts were more prominently molluscicidal than other extracts, with the methanol extract outperforming the rest (Aziz et al. 2021).

These studies suggest the usefulness of *T. crispa* as a biopesticide. Bio-assay guided isolation and analysis of active compounds should be carried out in the future in order to discover new natural chemical entities that could replace the harmful commercial pesticides currently used.

## Clinical trials

The clinical trials conducted thus far with *T. crispa* have focused entirely on the assessment of its antidiabetic properties (Table 4). One placebo-controlled, double-blind, randomized trial was conducted on 20 type-2 diabetic patients who were non-responsive to oral antidiabetic drugs and did not receive insulin. Following administration of *T. crispa* (1 g dry powder thrice a day for 6 months), no significant differences were observed between the *T. crispa*-treated group and the control group in terms of fasting blood sugar, insulin, and glycosylated hemoglobin levels. Unexpectedly, the *T. crispa*-treated group displayed higher cholesterol and glycosylated hemoglobin concentrations. Interestingly, an average of 2 kg of body weight loss was observed among the treated patients (Sangsuwan et al. 2004). One placebo-controlled, double-blind, randomized, crossover study conducted on 36 patients with metabolic syndrome revealed that treatment with *T. crispa* (250 mg capsules daily for two months) significantly lowered fasting blood sugar and triglyceride levels, but induced hepatotoxicity with ALT and AST levels noticeably increased in about 16.7% of the patients (Sriyapai et al. 2009). Another trial, conducted in Thailand, showed that *T. crispa* administered as a single dose (6 g) to non-diabetic healthy volunteers neither induced acute changes in glucose metabolism nor significantly improve glucose tolerance in 9 subjects. A similar single 6 g dose administered to 6 different healthy volunteers led to a significant decrease in blood glucose levels, but no changes in insulin levels. To check the biochemical and hematological effects of the plant, 12 subjects were treated with *T. crispa* 1 g thrice daily for 8 weeks while 13 others received 1.05 g doses in a similar fashion. Serum glucose and other hematological parameters were unchanged, except for AST and ALT levels which were noticeably increased, indicating hepatotoxicity (Rattanajarasroj et al. 2004). A more recent study to observe the effects of *T. crispa* ingestion employed 10 healthy and 10 diabetic subjects. The subjects received 75 g of glucose with or without 250 mg of *T. crispa* supplements after overnight fasting, and serum samples were collected every 30–60 min for 3 h. No significant changes in glucose or insulin levels were observed between the control and test groups (Klangjareonchai and Roongpisuthipong 2012).

**Table 4:** Clinical studies involving *Tinospora crispa*

Administered material	Number of Subjects	Health condition of subjects	Dosage regimen	Findings	Reference
Plant powder in capsules	20	Type-2 diabetic patients	1 g thrice daily for 6 months	No significant differences in terms of fasting blood sugar, insulin, and glycosylated haemoglobin levels. * Tinospora crispa*-treated group displayed higher cholesterol and glycosylated haemoglobin levels. Weight loss (average of 2 kg of body weight) was commonly observed	(Sangsuwan et al. 2004)
Plant powder in capsules	36	Patients with metabolic syndrome	250 mg twice daily for 2 months	Significantly lowering of fasting blood sugar and triglyceride levels, increase of AST and ALT	(Sriyapai et al. 2009)
Plant powder in capsules	9	Healthy	4 or 6 g, single dose	Did not improve glucose tolerance significantly	(Rattanajarasroj et al. 2004)
	6	Healthy	6 g, single dose	No changes in insulin levels, but significant decrease in blood glucose levels	
	12	Healthy	1 g thrice daily for 8 months	Unchanged hematological parameters, increased levels of AST and ALT	
	13	Healthy	1.05 g thrice daily for 4 months	Unchanged hematological parameters, increased levels of AST and ALT	
Plant powder in capsules	10	Healthy	125 or 250 mg, single dose	No significant changes in glucose or insulin levels	(Klangjareonchai and Roongpisuthipong 2012)
	10	Diabetic	125 or 250 mg, single dose	No significant changes in glucose or insulin levels	

The clinical trials carried out so far are preliminary with small sample size and non-systematic. To evaluate the plant as a safe and effective antidiabetic agent for human use, a thorough, serious and more operationally randomized controlled trial have to be performed.

## Safety and toxicological profile

Many studies have indicated that *T. crispa* extracts are relatively safe for oral ingestion. However, some studies have highlighted the hepatotoxicity potential of this plant. The ethanol extract of *T. crispa*, administered at a dose of 100–200 mg/kg, has displayed dose-dependent hepatotoxicity in thioacetamide-conditioned Sprague Dawley rats. The extract caused significant increases in the serum levels of ALT, AST, AP, bilirubin, and G-glutamyl transferase, and histological features of hepatocytic degeneration were also observed (Kadir et al. 2011). Similar elevation of AST and ALT were also reported in two Thai clinical studies involving *T. crispa* (Sriyapai et al. 2009; Rattanajarasroj et al. 2004). Two cases of toxic hepatitis following the use of *T. crispa* have been reported to date. The first one was a 49-year-old male who had been using a *T. crispa*-containing herbal medication (Langrand et al. 2014). The second was a 57-year-old man who ingested the aqueous extract of the plant (Cachet et al. 2018).

Clerodane-type furanoditerpenoids and borapetosides have been suggested as the constituents responsible for the observed in vivo toxicity of *T. crispa*. However, one study using a LPS-induced ND-4 mice model reported that borapetosides B (**17**), C (**14**), and F (**25**) did not produce hepatotoxicity, when administered both individually and in combination at a dose of 500 mg/kg for 21 days (Parveen et al. 2020). *Tinospora crispa* ethanol extract, and the *n*-hexane and chloroform fractions from its methanol extract, have been found to be quite safe in murine models. No harmful effects were observed neither following the administration of the ethanol extract (50–200 mg/kg) to male Balb/C mice, nor following the administration of fractions from the methanol extract (various doses with the maximal dose of 2000 mg/kg) on Swiss albino mice (Ahmad et al. 2016b; Rakib et al. 2020a). A dermal irritation test employing adult albino rabbits showed that a *T. crispa*-based ointment was non-irritant when administered topically (Torre et al. 2017).

Potential drug-drug interactions have been suggested between *T. crispa* and other co-administered drugs through its capacity of modulating the Pregnane X-receptor (PXR). In an in vitro luciferase reporter gene assay, the methanol extract of the plant and its chloroform and *n*-hexane fractions significantly activated PXR. Its ethyl acetate and the butanol fractions showed negligible activity. Several *T. crispa* constituents shared this activity, and the in vitro results were further reflected in silico (Parveen et al. 2022). Thus, any drug being metabolized or activated through PXR might experience altered pharmacokinetics when co-administered with *T. crispa*. Given the potential of *T. crispa* as a source for novel therapeutic lead compounds, further comprehensive studies should be conducted to establish its absolute safety.

## Conclusion and future prospects

Multiple in vitro and in vivo studies on *T. crispa* have demonstrated its remarkable medicinal potential, particularly in the treatment of diabetes and hypertension, providing support to justify some of its ethnobotanical uses. Several clerodane-type furanoditerpenoids in *T. crispa* have been reported to possess significant antidiabetic activity, which is worthy of further exploration for the discovery of novel antidiabetic drugs. In addition, the adrenergic activity of its alkaloids may provide new avenues for the treatment of high blood pressure. While the cytotoxic prospects of this plant have been studied at length, the specific molecular mechanisms involved in this effect have yet to be elucidated. Clerodane-type furanoditerpenoids have revealed noticeable results as chemopreventive agents, which is also worthy of further investigations. The widespread use of *T. crispa* as an antimalarial agent has been supported by many studies, but with conflicting reports on its efficacy. It is interesting to note, however, that various extracts provide protection against malaria, which may help to offset the detrimental effects of this disease. These observations suggest the need for further bioassay-guided isolation in order to identify the antiplasmodial phytoconstituent(s) of *T. crispa*. Likewise, the immunomodulatory activity of various *T. crispa* extracts and phytoconstituents demands scrutiny. One of the most notable aspects in this regard is the ability of *T. crispa* phytoconstituents to both activate and suppress the immune system. Alkaloids from *T. crispa* have been found to be particularly potent and deserve closer inspection. Among the other pharmacological effects displayed by the plant, the inhibition of acetylcholinesterase by quaternary alkaloids looks promising and should be investigated further. Although the hepatotoxicity of *T. crispa* has been attributed to the presence of selected clerodane-type furanoditerpenoids, there are other compounds within that class that are not hepatotoxic. Therefore, in-depth investigations into this class of phytochemicals are essential in order to evaluate their relative safety and toxicity. The characteristic phytoconstituents of *T. crispa* may overall play an important part in the discovery of new drug leads. In that respect, additional in vivo pharmacological and toxicological studies on *T. crispa* are warranted to provide assurance of adequate efficacy and safety.

## Data Availability

Not applicable.
